# Neuroprotective Nanoplatform Integrating Antioxidant MXene Nanozymes and Ferroptosis Inhibitors for Targeted Therapy of Cerebral Ischemia‐Reperfusion Injury

**DOI:** 10.1002/advs.202516001

**Published:** 2025-11-21

**Authors:** Lu Wang, Chao Hou, Shuo Li, Lizhi Yang, Yiqun Lin, Sen Wang, Xiaohua Jia, Hui Hui, Wen He, Wei Zhang

**Affiliations:** ^1^ Department of Ultrasound Beijing Tiantan Hospital Capital Medical University Beijing 100070 China; ^2^ Key Laboratory of Molecular Imaging of Chinese Academy of Sciences Institute of Automation Chinese Academy of Sciences Beijing 100190 China

**Keywords:** cerebral ischemia‐reperfusion injury, inflammatory microenvironment remodeling, MXene nanozymes, neuronal ferroptosis, oxidative stress

## Abstract

Even after successful revascularization in acute ischemic stroke, some patients still develop secondary neuronal damage and functional impairment, known as cerebral ischemia‐reperfusion injury (CIRI). The complex pathophysiological cascade within CIRI limits the efficacy of current single‐target therapies in clinical practice. To address this, this study innovatively constructs a multifunctional brain‐targeted nanoplatform (RFP) designed to synergistically intervene in the multiple pathological pathways of CIRI. This platform co‐delivers Pt‐based MXene nanozymes (Pt‐Ti_3_C_2_) with SOD/CAT‐like activity and the ferroptosis‐specific inhibitor (Fer‐1), enabling targeted delivery to the cerebral ischemic region via surface‐modified cRGD peptides. The Pt‐Ti_3_C_2_ nanozyme core within the RFP exhibits SOD/CAT‐like enzymatic activity, efficiently scavenging multiple ROS to inhibit oxidative stress. Meanwhile, Fer‐1 embedded within the lipid bilayer suppresses lipid peroxidation, thereby blocking ferroptosis. Their synergistic action further mitigates neuroinflammation by suppressing pathological glial activation. In vivo and in vitro studies have demonstrated that RFP exhibits outstanding specific targeting efficacy and significant neuroprotective effects. Multi‐omics analysis reveals that RFP exerts multi‐effect therapy by synergistically suppressing oxidative stress and ferroptosis, maintaining mitochondrial homeostasis, and modulating inflammatory networks. This multifunctional nanotherapeutic strategy represents a shift from single‐target treatment to multi‐mechanism synergy, offering a novel approach to overcome CIRI therapeutic challenges.

## Graphical abstract

Cerebral ischemia‐reperfusion injury (CIRI) is a key pathological process limiting the efficacy of stroke therapy. This study has developed an innovative, multifunctional, brain‐targeted nanoplatform (RFP) containing MXene nanoenzymes and ferroptosis inhibitors. It intervenes synergistically in the cascading pathological processes of oxidative stress, ferroptosis, and neuroinflammation to achieve multi‐effect treatment of CIRI. This study presents a novel approach for treating CIRI.

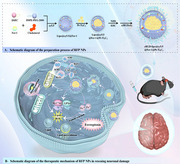

## 1 Introduction

Acute ischemic stroke is a serious disease that endangers human life and health, characterized by high incidence, disability, and mortality rates.^[^ ^1^, ^2^ ^]^ Clinically, thrombolysis or thrombectomy can rapidly restore blood supply to the ischemic side of the brain. However, even with early restoration of blood supply, patients may still experience secondary neuronal damage and functional impairments, known as cerebral ischaemia‐reperfusion injury (CIRI).^[^ ^3^, ^4^ ^]^ Therefore, how to precisely target CIRI is an important research direction for improving efficacy and reducing patient mortality and disability rates.

CIRI involves complex pathophysiological cascades.^[^ ^5^, ^6^ ^]^ During reperfusion, as large amounts of oxygen and nutrients flood into the cells, Mitochondria, as the cellular centers for energy metabolism and oxidative stress regulation, attempt to help damaged cells restore normal function. In this process, excessive reactive oxygen species (ROS) are produced, triggering oxidative stress reactions.^[^ ^7^, ^8^ ^]^ Concurrently, iron homeostasis imbalance leads to intracellular Fe^2^⁺ accumulation. Specifically, H_2_O_2_ reacts with Fe^2^⁺ via the Fenton reaction to generate highly reactive hydroxyl radicals (·OH), which attack polyunsaturated fatty acids in the cell membrane, triggering lipid peroxidation. When the antioxidant defense system is inactivated, accumulated lipid peroxides induce ferroptosis, disrupting membrane integrity and causing ion imbalance and cell death.^[^ ^9^, ^10^, ^11^, ^12^ ^]^ Neurons undergoing ferroptosis release damage‐associated molecular patterns (DAMPs), which activate microglia. ROS also act as pro‐inflammatory signals, further exacerbating neuroinflammation. Ultimately, this forms a vicious cycle of oxidative stress‐ferroptosis‐neuroinflammation, leading to the occurrence and development of cerebral ischaemia‐reperfusion injury.^[^ ^13^, ^14^, ^15^, ^16^ ^]^ Previous studies have focused on neuroprotective research targeting single molecular mechanisms, including the use of antioxidants to inhibit ROS or anti‐inflammatory drugs to regulate microglia, all of which can intervene in CIRI to a certain extent.^[^ ^17^, ^18^, ^19^, ^20^ ^]^ However, due to issues such as low blood‐brain barrier permeability and the failure to block multi‐pathway collaboration, the application of single‐target drugs in clinical settings still faces many challenges and limitations. This underscores the importance and urgency of shifting research from single‐target to multi‐effect therapies.^[^ ^21^, ^22^ ^]^ Therefore, developing a multifunctional nanoplataform that simultaneously targets oxidative stress, ferroptosis, and neuroinflammation is a key strategy to overcome clinical treatment bottlenecks.

In recent years, the emergence of biomimetic nanozymes has provided innovative strategies for intervening in oxidative stress. Compared to traditional antioxidants, nanoenzymes not only possess controllable catalytic activity and excellent environmental tolerance but also demonstrate significant advantages in stability, sustainability, and therapeutic efficacy.^[^ ^23^, ^24^ ^]^ Specifically, traditional small‐molecule antioxidants (such as edaravone) exhibit short in vivo half‐lives, lack targeting specificity, and can only transiently scavenge reactive oxygen species through stoichiometric reactions.^[^ ^25^ ^]^ Meanwhile, natural enzyme preparations face inherent limitations, including complex extraction processes, poor stability, and susceptibility to protease degradation.^[^ ^26^ ^]^ In contrast, nanozymes, through their stable inorganic material substrates and designable catalytic active centres, not only emulate the catalytic functions of natural enzymes but also achieve prolonged, sustainable ROS scavenging. Furthermore, rational material design enables the incorporation of targeted delivery capabilities, thereby substantially surpassing conventional antioxidant therapies in both therapeutic efficacy and practical utility.^[^ ^27^, ^28^, ^29^ ^]^ 2D transition metal carbides (MXene) are a new class of 2D layered materials. Among them, Ti_3_C_2_ MXene exhibits exceptional catalytic potential due to its unique layered structure, high conductivity, and surface‐rich ─OH/─O active groups.^[^ ^30^, ^31^, ^32^, ^33^ ^]^ In particular, the platinum‐loaded Ti_3_C_2_ MXene system (Pt‐Ti_3_C_2_ MXene) can simulate SOD/CAT cascade activity through the synergistic effect of the Pt crystal plane and the MXene substrate, achieving efficient removal of reactive oxygen species such as •O_2_^―^/H_2_O_2_.^[^ ^34^ ^]^ However, single ROS scavenging is unable to block the chain accumulation of lipid peroxides (LPO) in the core pathway of ferroptosis, requiring intervention with specific inhibitors. Ferrostatin‐1 (Fer‐1), as an inhibitor of ferroptosis, efficiently quenches lipid free radicals (LOO·) through a hydrogen atom transfer mechanism, protecting neurons from membrane lipid peroxidation damage and intervening in the occurrence and development of ferroptosis.^[^ ^35^ ^]^ Pt‐Ti_3_C_2_ MXene and Fer‐1 can reduce pathological activation of glial cells, thereby inhibiting neuroinflammation and ultimately blocking the cascade of oxidative stress‐ferroptosis‐inflammatory response. Therefore, the development of such multi‐functional nanoplatforms is of great value in curbing cerebral ischaemia‐reperfusion injury.

**Scheme 1:**
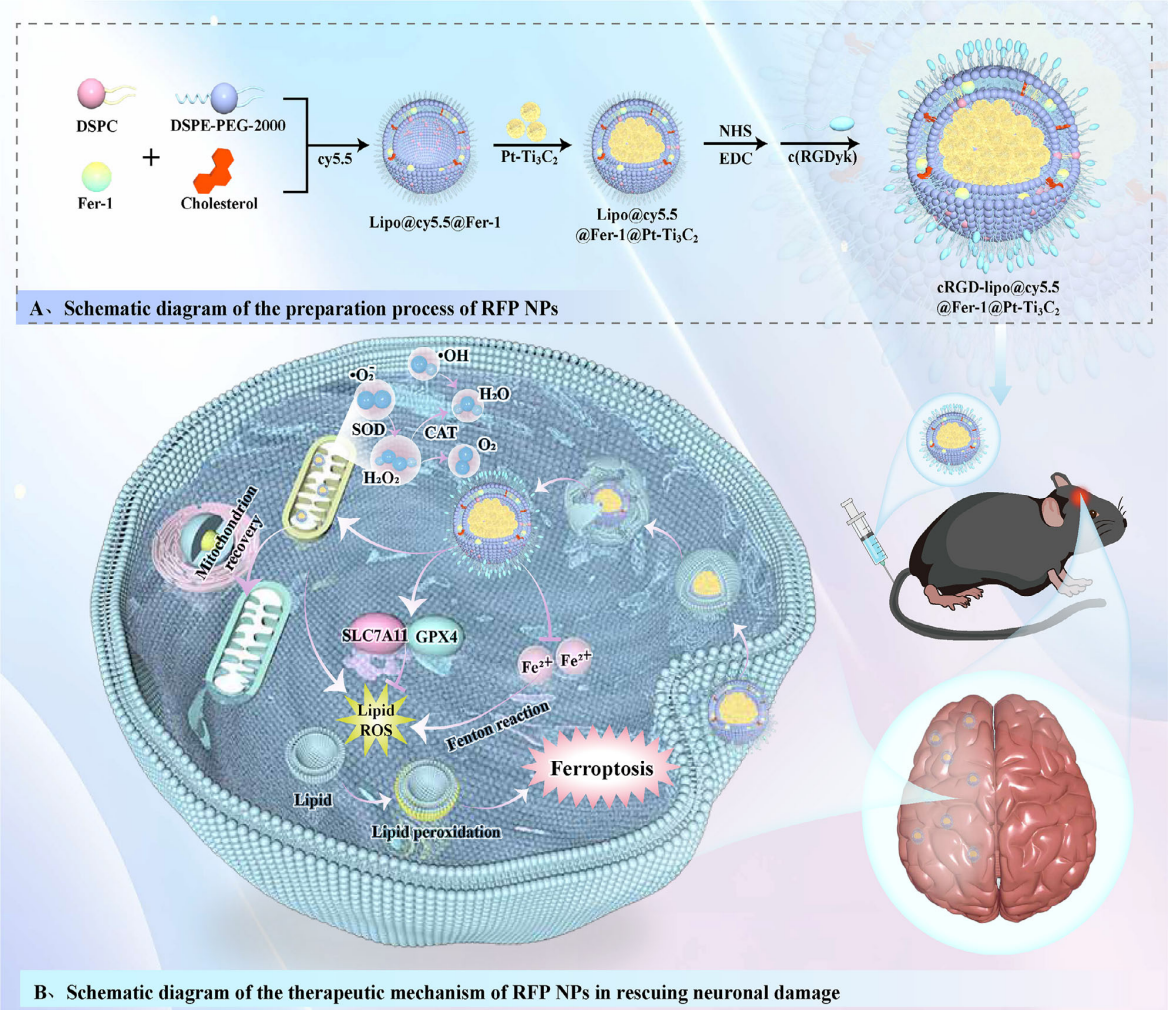
RFP preparation process and therapeutic mechanism diagram. A) Schematic diagram of the preparation process of RFP NPs. B) Schematic diagram of the therapeutic mechanism of RFP NPs in rescuing neurons by remodelling the damaged microenvironment.

Although Pt‐Ti_3_C_2_ MXene and Fer‐1 both show therapeutic potential in intervening in CIRI, their combined application still faces challenges such as low brain targeting efficiency and component interference. How to construct them as a whole to achieve synergistic therapeutic effects and how to target delivery to the ischaemic brain injury area are important issues that need to be addressed. Therefore, we constructed a therapeutic system co‐loaded with Pt‐Ti_3_C_2_ and Fer‐1 using cRGD‐functionalized liposomes (RFP NPs). The surface‐modified c(RGDyK) peptide can specifically bind to the integrin αvβ3 overexpressed in the brain ischaemic area, which enables precise delivery to the lesion site, enhances drug accumulation, and reduces systemic toxicity. The design of the lipid carrier involves embedding Fer‐1 into a hydrophobic bilayer and encapsulating Pt‐Ti_3_C_2_ into a hydrophilic core, thereby achieving spatial separation to avoid component interference and maintain the activity of each drug. Utilising the catalytic properties of Pt‐Ti_3_C_2_ to simulate SOD/CAT cascade activity, achieving the purpose of removing ROS and alleviating oxidative stress. Utilising Fer‐1 to inhibit lipid peroxidation protects cells from ferroptosis damage and indirectly reduces the recruitment and activation of glial cells, thereby synergistically inhibiting neuroinflammation. In summary, this study innovatively developed a multifunctional brain‐targeted nanotherapeutic system that can effectively remodel the complex microenvironment of CIRI lesions, providing a new strategy for solving the treatment challenges of CIRI (**Scheme** **1**).

## 2 Results and Discussion

### 2.1 Preparation and Characterisation of Nanoscale Systems

**Figure 1:**
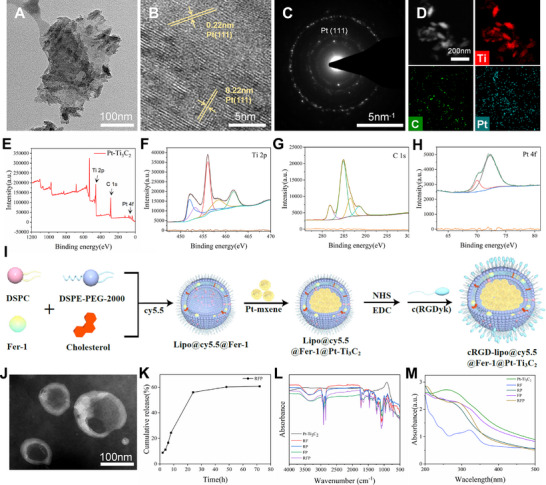
Synthesis and characterisation. A) TEM image of Pt‐Ti_3_C_2_ (scale bar, 100 nm). B) HRTEM image of Pt‐Ti_3_C_2_ (scale bar, 5 nm). C) SAED pattern of Pt‐Ti_3_C_2_ (scale bar, 5 nm^−1^). D) EDS mapping of Pt‐Ti_3_C_2_ (scale bar, 200 nm). E) XPS pattern of Pt‐Ti_3_C_2_. F–H) XPS patterns of Ti(F), C(G), and Pt(H) in Pt‐Ti_3_C_2_. I) Schematic diagram of the preparation process for RFP NPs. J) TEM image of RFP NPs (scale bar, 100 nm). K) Fer‐1 cumulative release curve of RFP NPs. L) FTIR spectrum of RFP NPs. M) UV–Vis spectrum of RFP NPs.

Firstly, we successfully synthesized Pt‐Ti_3_C_2_ MXene via a fluoride salt/strong acid etching method coupled with an in situ reduction approach. Transmission electron microscopy (TEM) and scanning electron microscope (SEM) confirmed that the material retained a typical 2D layered structure with extremely small platinum (Pt) nanoparticles (NPs) uniformly loaded on the surface of the Ti_3_C_2_ MXene substrate (**Figure** **1**A; Figure S1, Supporting Information). High‐resolution TEM (HRTEM) showed that the lattice spacing was ≈0.22 nm, corresponding to the Pt(111) crystal plane (Figure 1B). Selected area electron diffraction (SAED) confirmed that Pt NPs exist in a face‐centred cubic (fcc) crystal structure, forming a clear interface with the Ti_3_C_2_ MXene substrate without phase transformation (Figure 1C). The Pt(111) plane typically exhibits the highest atomic density and catalytic activity. The above results indicated that Ti_3_C_2_ MXene exposed a large amount of highly catalytic surface, which was significant for its application as a nanozyme for scavenging ROS.EDS mapping showed the distribution of Ti, C, and Pt elements in the layered structure (Figure 1D). Quantitative EDS analysis showed that the Pt loading was 2.42 wt.%, which is a moderate loading that ensures sufficient catalytic active site density while avoiding the risk of Pt NPs agglomeration that may result from excessive loading (Figure S2, Supporting Information). X‐ray Photoelectron Spectroscopy (XPS) confirmed that the nanomaterial is primarily composed of Ti, C, Pt, and O elements. The 455.0 eV characteristic peak (Ti‐C bond) in the Ti 2p spectrum and the 282.0 eV peak in the C 1s spectrum jointly validated the integrity of the MXene framework, ensuring the material's conductivity and mechanical stability. The Pt 4f double peak (71.0/74.3 eV) indicated that Pt exists primarily in its metallic state (Pt⁰), providing the foundation for catalytic activity (Figure 1E–H). The results found that the peak positions of Ti 2p and C 1s may be slightly shifted, indicating that there is electron transfer between Pt NPs and Ti_3_C_2_ Mxene. This electron transfer is conducive to enhancing the interface bonding strength and synergistic catalytic effect. X‐Ray Diffraction (XRD) showed a peak at 9°–10°(002) reflecting the layered order of MXene. The 39.8° diffraction peak in XRD corresponded to the (111) crystal plane of fcc‐Pt, which corresponded to the diffraction ring observed in SAED. Both confirmed the fcc crystal structure of Pt (Figure S3, Supporting Information).

To synergistically inhibit oxidative stress and ferroptosis while achieving brain‐targeted delivery, this study innovatively integrated a 2D MXene nanozyme with the small‐molecule ferroptosis inhibitor Fer‐1 into a single nanodelivery system. This specific synergistic combination has not been previously reported. Spatial separation of the two functional components was achieved by embedding hydrophobic Fer‐1 within the lipid bilayer while encapsulating hydrophilic Pt‐Ti_3_C_2_ in the liposomal core. This design prevented potential mutual interference and preserved their respective bioactivities. The synthetic methodology employed in this study was based upon and optimized from classical liposome preparation techniques, thereby ensuring the reproducible fabrication of the nanocarriers.^[^ ^36^, ^37^ ^]^ The constructed liposomal systems included the following formulations: Fer‐1‐loaded liposomes (RF), Pt‐Ti_3_C_2_‐loaded liposomes (RP), non‐targeted dual‐loaded liposomes (FP), and fully functional cRGD‐targeted liposomes (RFP) (Figure 1I). TEM and SEM showed that all liposomes had a nearly spherical morphology and intact bilayer structure, with no obvious drug leakage, membrane rupture, or aggregation observed (Figure 1J; Figures S4 and S5, Supporting Information). To evaluate the consistency and reproducibility of our synthesis process, all four types of nanoparticles (RF, RP, FP, and RFP) were synthesized and characterized across multiple batches. Dynamic light scattering (DLS) results indicated that all batches exhibited highly uniform size distributions and stable zeta potentials. Taking the core system RFP as an example, the average hydrodynamic size was (191.33 ± 4.16) nm with a zeta potential of (−30.87 ± 0.64) mV (Figure S6 and Table S1, Supporting Information). These results demonstrate that the preparation method employed in this study offers excellent reliability and reproducibility. The Fer‐1 encapsulation rate of RFP NPs was 85.5%, with a drug loading rate of 10.18% (Figure S7, Supporting Information). The in vitro cumulative release curve exhibited a biphasic characteristic, with 55% release within 0–24 h and a cumulative release of 60% at 72 h (Figure 1K). This release profile, which combines a rapid onset of action with prolonged maintenance, provides an excellent foundation for drug release intervention in CIRI. The effective modification of the cRGD peptide and the successful loading of Fer‐1/Pt‐Ti_3_C_2_ were validated using Fourier transform infrared spectroscopy (FTIR) and Uv–vis spectroscopy (UV–vis). The presence of the amide I band at 1650 cm^−1^ in FTIR confirmed the complete modification of cRGD (Figure 1L). UV–vis revealed that the characteristic absorption peak of Fer‐1 at 280 nm coexists with the broad absorption band of Pt‐Ti_3_C_2_ at 200–300 nm (Figure 1M), confirming the spatial separation of the two components within the liposome. The results of nanoparticle stability studies indicated that RFP remained stable for up to 72 h when incubated in PBS or DMEM supplemented with 10% foetal bovine serum (Figure S8, Supporting Information).

### 2.2 Evaluation of In Vitro SOD/CAT‐Like Enzyme Activity of Pt‐Ti_3_C_2_ MXene

**Figure 2:**
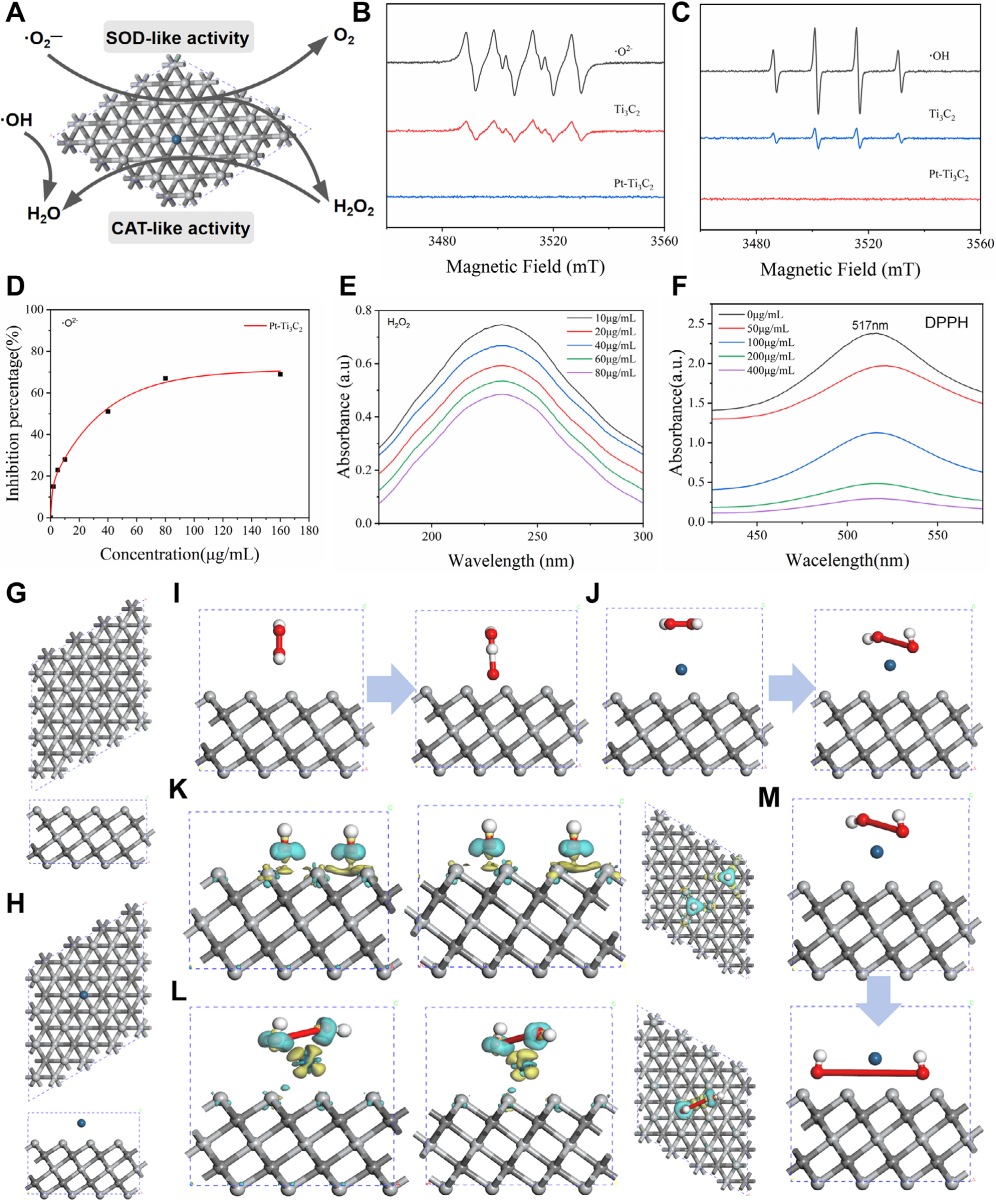
SOD‐like/CAT‐like enzyme activity of Pt‐Ti_3_C_2_ MXene. A) Schematic diagram of the catalytic activity of Pt‐Ti_3_C_2_ MXene nanoenzymes. B,C) ESR spectra analysis of the scavenging ability of Pt‐Ti_3_C_2_ MXene against •O_2_^―^(B) and •OH(C) in vitro. D,E) Quantitative characterisation results of Pt‐Ti_3_C_2_ with SOD‐like enzyme activity (D) and CAT‐like activity (E). F) Quantitative analysis results of the DPPH scavenging ability of Pt‐Ti_3_C_2_ in vitro. G,H) Structural diagrams of Ti_3_C_2_ (G) and Pt‐Ti_3_C_2_ (H) (upper: top view; lower: side view). I,J) Structural diagrams of Ti_3_C_2_ (I) and Pt‐Ti_3_C_2_ (J) before and after H_2_O_2_ adsorption (left: before adsorption; right: after adsorption). K,L) Differential charge density analysis of Ti_3_C_2_ (K) and Pt‐Ti_3_C_2_ (L). Yellow represents electron consumption, and cyan represents electron accumulation. M) Structural diagrams of the initial state and transition state of Pt‐Ti_3_C_2_ after H_2_O_2_ adsorption (upper: initial state; lower: transition state).

Excessive ROS is a key factor in triggering oxidative stress and ferroptosis. In Pt‐Ti_3_C_2_ MXene nanoenzymes constructed in this study, the fcc crystal structure of Pt NPs provides abundant active sites, while the Ti_3_C_2_ MXene substrate synergistically enhances ROS scavenging efficiency through surface functional groups and the conductivity of its 2D structure (**Figure** **2**A). Electron spin resonance (ESR) confirmed the radical scavenging ability of Pt‐Ti_3_C_2_ MXene (Figure 2B,C). •O_2_^―^ is the “initiator” of radical chain reactions and the main target of the SOD enzyme.^[^ ^38^ ^]^ In Figure 2B, the control group exhibited typical •O_2_^―^ quadruple peak signals. After adding Ti_3_C_2_ and Pt‐Ti_3_C_2_, respectively, it was found that the Pt‐Ti_3_C_2_ had a significantly greater ability to scavenge •O_2_^―^ than the Ti_3_C_2_ (the signal intensity dropped to near baseline), proving that adding Pt nanoparticles greatly enhances the material's SOD‐like activity. Hydroxyl radicals (•OH) are the most oxidizing reactive oxygen species and key drivers of ferroptosis. Figure 2C showed that the control group exhibits the typical 1:2:2:1 •OH peak. Compared to the Ti_3_C_2_ treatment group, the signal intensity was significantly suppressed after Pt‐Ti_3_C_2_ MXene treatment, indicating the •OH scavenging ability of Pt‐Ti_3_C_2_ MXene nanoenzymes. Further enzyme kinetic studies showed that the SOD‐like activity of Pt‐Ti_3_C_2_ MXene exhibited a rapid dose response in the concentration range of 10–40 µg mL^−1^, demonstrating high catalytic efficiency and strong substrate affinity (Figure 2D). At 40 µg mL^−1^, the inhibition rate of •O_2_^―^ reached 50%. As the concentration gradually increased to 160 µg mL^−1^, the inhibition rate reached 70% and approached equilibrium, consistent with the classical Michaelis–Menten enzyme saturation kinetics.^[^ ^39^ ^]^ The CAT‐like activity is manifested by the systematic decrease in the characteristic peak of H_2_O_2_ at 240 nm with increasing Pt‐Ti_3_C_2_ MXene concentration (10–80 µg mL^−1^), confirming its high efficiency in decomposing H_2_O_2_ (Figure 2E). DPPH is a stable organic radical. The results of the DPPH scavenging experiment showed that the absorbance at 517 nm decreased with increasing Pt‐Ti_3_C_2_ concentration (50–400 µg mL^−1^), with a scavenging rate >90% at 400 µg mL^−1^ (Figure 2F). The above results confirmed the high scavenging efficiency of Pt‐Ti_3_C_2_ MXene for •O_2_^―^, •OH, H_2_O_2_, and DPPH. This broad‐spectrum antioxidant ability provided a molecular basis for the synergistic blocking of the oxidative stress‐ferroptosis cascade with Fer‐1.

Based on density functional theory (DFT) calculations, this study revealed the catalytic mechanism of Pt‐Ti_3_C_2_ MXene for efficient removal of reactive oxygen species at the atomic scale. It was found that Pt atoms were stably loaded at the centre of the Ti_3_C_2_ hexagonal ring to form a composite structure (Figure 2G,H). Its geometric steric hindrance and surface reconstruction effect optimized the adsorption energy of H_2_O_2_ from the strong chemical adsorption (−3.66 eV) of Ti_3_C_2_ to −1.44 eV (Figure 2I,J), avoiding catalyst site poisoning and complying with the Sabatier principle. The differential charge density of Pt‐Ti_3_C_2_ (0.84 e) was increased compared to Ti_3_C_2_ (0.77 e), indicating that the charge transfer between the Pt‐modified system and H_2_O_2_ molecules was more significant (Figure 2K,L). This enhanced charge transfer is primarily attributed to the electron‐donating effect of Pt atoms and electrostatic induction effects, indicating that Pt‐Ti_3_C_2_ can more effectively activate H_2_O_2_ molecules and promote their decomposition reactions. The density of states (DOS) spectrum confirmed that the modification of Pt did not alter the intrinsic electronic structure characteristics of Ti_3_C_2_. The total DOS and projected DOS (PDOS) distributions of Ti_3_C_2_ and Pt‐Ti_3_C_2_ nearly overlap (Figure S9, Supporting Information). The consistency between DOS and PDOS indicated that the interaction between Pt atoms and Ti_3_C_2_ was relatively weak, primarily involving physical adsorption or weak chemical interactions rather than strong covalent bonding. This “mild” modification approach preserved the excellent intrinsic electronic properties of Ti_3_C_2_ while precisely regulating H_2_O_2_ adsorption behaviour through geometric effects and local environment modulation. Transition state calculations indicated that Pt was the primary catalytic active center in the Pt‐Ti_3_C_2_ system. In the initial stage of the reaction, H_2_O_2_ bound to the Pt active site via the O atom. As the reaction progressed to the transition state, the O─O bond elongated and weakened before breaking. This resulted in significant geometric distortion of H_2_O_2_, with the system reaching an energy saddle point (Figure 2M). The activation energy for this catalytic process is extremely low (0.0098 eV, ≈0.225 kcal mol^−1^), confirming its catalytic properties with an energy barrier approaching zero. The above DFT analysis comprehensively illustrated the atomic mechanism of efficient ROS removal by Pt‐Ti_3_C_2_ MXene from the perspectives of adsorption regulation, charge transfer, and ultra‐low energy barrier. These results provide a theoretical basis for treating cerebral ischemia‐reperfusion injury.

### 2.3 Assessment of the Ability of RFP to Inhibit Oxidative Stress and Ferroptosis In Vitro

**Figure 3:**
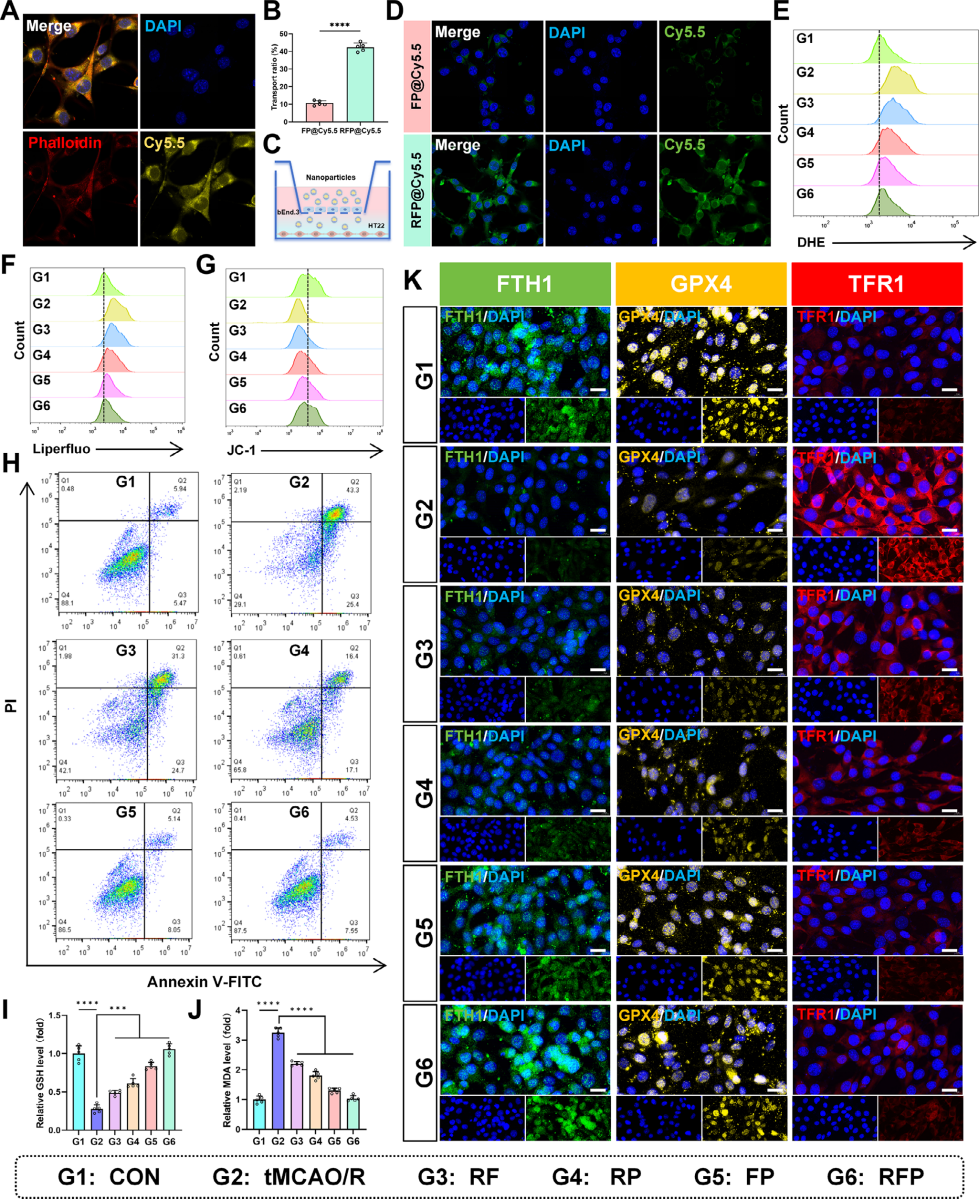
RFP protects cells by clearing ROS from HT22 cells and inhibiting ferroptosis. A) Cell uptake images after co‐incubation of RFP and HT22 cells for 30 min (1000X). B) Penetration percentage of free FP@Cy5.5 and RFP@Cy5.5 through the in vitro BBB model. Calculation of penetration percentage was performed by normalizing the fraction of formulations that penetrated blank filter inserts. Results are reported as means ± SD, n = 5. C) Illustration of the in vitro BBB transwell model. D) Confocal images of the HT22 cells in the bottom compartment after incubation with different nanoparticles for 24 h (600X). (E) Flow cytometry results for ROS levels in each group of cells. F) Flow cytometry results for lipid peroxide levels in each group of cells. G) Flow cytometry results of mitochondrial membrane potential levels in each group of cells. H) Flow cytometry results for apoptosis levels in each group of cells. (I) Results of GSH content detection in each group of cells. Results are reported as means ± SD, n = 5. J) Results of MDA content detection in each group of cells. Results are reported as means ± SD, n = 5. K) Representative images of immunofluorescence staining for ferroptosis‐related proteins FTH1, GPX4, and TFR1 in each group of cells (scale bar, 20 µm). ^***^ *p* <0.001, ^****^ *p* <0.0001.

First, we tested the cytotoxicity of RFP using CCK‐8. After incubating HT22 cells with different concentrations of RFP for 24 h, no significant inhibition of cell activity was observed, indicating that the nanoparticles we synthesized have good biocompatibility (Figure S10, Supporting Information). To investigate the uptake characteristics of RFP in HT22 cells, we prepared RFP@Cy5.5. After incubating 10 µg mL^−1^ of RFP@Cy5.5 with HT22 for 30 min, we observed a significantly enriched fluorescence signal of the nanoparticles in the cells through confocal microscopy (**Figure** **3**A). Following the demonstration of efficient cellular uptake of RFP@Cy5.5 in HT22 cells, we next sought to investigate the ability of this targeted nanomedicine to cross the blood‐brain barrier (BBB), which is crucial for any neurological therapeutic application. We first successfully established an in vitro BBB model using bEnd.3 cells, with its integrity verified by monitoring transendothelial electrical resistance (TEER) (Figure S11A, Supporting Information). As shown in Figure S11B (Supporting Information), the TEER values increased steadily during culture and eventually stabilized above 200 Ω·cm^2^. This value was established as the threshold for a qualified model with intact barrier function, which was then used in all subsequent experiments. In permeability studies, we compared the transport efficiency between cRGD‐targeted RFP@Cy5.5 and non‐targeted FP@Cy5.5. The results showed that RFP@Cy5.5 achieved significantly higher translocation rates after 24 h, confirming that the cRGD peptide‐mediated active targeting mechanism effectively enhanced nanoparticle transport across the BBB (Figure 3B). To ensure this enhanced permeation was not due to cytotoxicity‐induced barrier damage, we performed CCK‐8 assays and verified that neither formulation significantly affected bEnd.3 cell viability, thereby excluding this possibility (Figure S11C,D, Supporting Information). Finally, to simulate the complete drug delivery pathway, we co‐cultured HT22 neuronal cells in the lower chamber of the BBB model and observed that RFP@Cy5.5 was more effectively internalized by HT22 cells (Figure 3C,D). These findings collectively demonstrate that cRGD modification not only facilitates BBB penetration but also enhances subsequent internalization by downstream neuronal cells, achieving a sequential “penetration–uptake” delivery process and highlighting its strong potential as a targeted intracerebral delivery platform.

This study used HT22 Oxygen‐Glucose Deprivation/Reperfusion (OGD/R) to simulate CIRI damage in vitro. The intense oxidative stress induced by OGD/R led to the massive production of ROS (especially from mitochondria) within cells. These excess ROS directly catalyzed iron‐dependent lipid peroxidation reactions, which were key upstream events in the initiation of ferroptosis. Therefore, we firstly detected the ROS levels in different treatment groups using flow cytometry. The results showed that compared with the normal group (G1), the ROS levels in the OGD/R group (G2) were significantly increased. Following intervention with RF (G3), RP (G4), FP (G5), and RFP (G6), ROS levels decreased to varying degrees. Among these, RFP NPs exhibited the strongest ROS scavenging activity, reducing ROS levels to near‐normal levels (Figure 3E; Figure S12, Supporting Information). Given that uncontrollable lipid peroxidation accumulation is a core marker of ferroptosis, we used the specific probe Liperfluo to detect each group. The results showed that lipid peroxidation products accumulated significantly in the OGD/R group cells (G2), while all four NPs reduced lipid peroxidation to varying degrees after treatment, with RFP NPs (G6) showing the most significant effect (Figure 3F; Figure S13, Supporting Information). During ferroptosis, excessive lipid peroxidation directly attacks the mitochondrial membrane, leading to mitochondrial structural abnormalities and functional failure. One of the core manifestations is a significant decrease in mitochondrial membrane potential (MMP). Therefore, we used JC‐1 probe detection and found that OGD/R treatment significantly reduced MMP, while the addition of different NPs significantly restored MMP. Similarly, RFP (G6) showed the most prominent ability to protect mitochondria (Figure 3G; Figure S14, Supporting Information). To assess the protective capacity of different NPs against OGD/R in HT22 cells, we used flow cytometry to detect the apoptosis levels in different groups. The results showed that all NPs had a significant protective effect, with RFP (G6) exhibiting the strongest cell protection capacity, consistent with the previous results (Figure 3H; Figure S15, Supporting Information). In addition, when oxidative stress and ferroptosis occur, glutathione (GSH) levels decrease and malondialdehyde (MDA) levels increase in OGD/R cells. We found that treatment with NPs can reverse these changes to varying degrees, significantly increasing intracellular GSH levels and decreasing MDA levels (Figure 3I,J). We also assessed the intracellular expression levels of key ferroptosis molecules and found that FTH1 and GPX4 were significantly decreased in OGD/R group cells (G2), while TfR1 was significantly increased. Treatment with NPs significantly reversed these changes, with RFP (G6) showing the most pronounced effect (Figure 3K; Figures S16 and S17, Supporting Information). In conclusion, in vitro studies have demonstrated that RFP exhibits excellent neuroprotective properties due to its exceptional ability to scavenge reactive oxygen species, protect mitochondria, and inhibit ferroptosis.

### 2.4 cRGD‐Modified RFP Nanoparticles Enable Efficient Blood‐Brain Barrier Penetration and Targeted Accumulation in Ischemic Hemisphere

**Figure 4:**
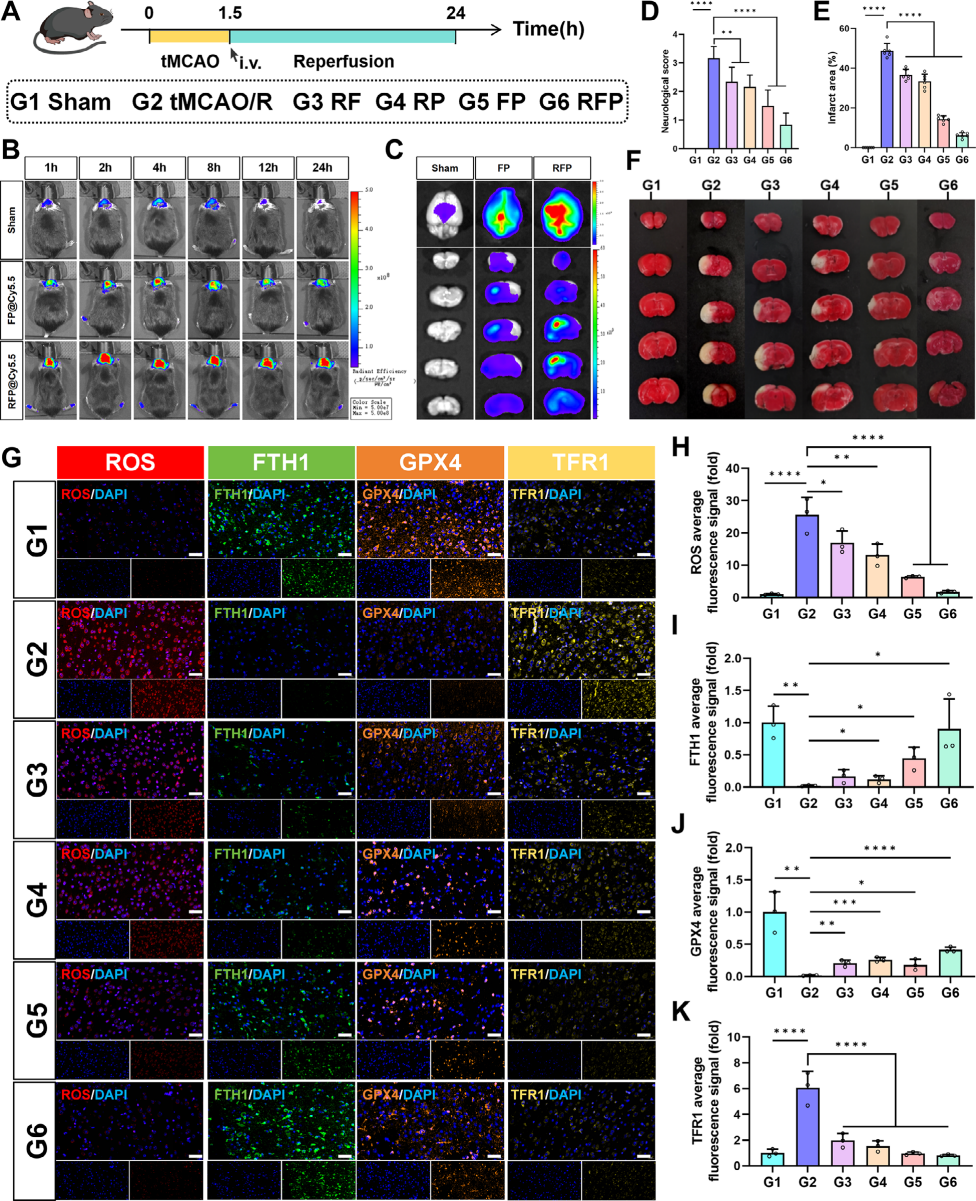
Therapeutic effects of RFP in tMCAO/R mice and its inhibitory effect on ferroptosis. A) Schematic diagram of tMCAO/R mouse model construction. B) Representative IVIS images of tMCAO/R model mice at different time points after injection of FP@Cy5.5 or RFP@Cy5.5. C) Representative IVIS images of brain and slices from tMCAO/R mice injected with FP@Cy5.5 or RFP@Cy5.5 for 24 h. D) The neurological score for each treatment group, n = 6. E,F) TTC brain section staining results for each group of mice and the quantitative analysis of infarct volume. Results are reported as means ± SD, n = 6. G) Representative images of immunofluorescence staining of ROS and ferroptosis‐related proteins FTH1, GPX4, and TFR1 in brain tissue from each group of mice (scale bar, 40 µm). H–K) Semi‐quantitative analysis of ROS (H), FTH1 (I), GPX4 (J), and TFR1 (K) immunofluorescence staining results. Results are reported as means ± SD, n = 3. ^*^ *p* <0.05, ^**^ *p* <0.01, ^***^ *p* <0.001, ^****^ *p* <0.0001.

**Figure 5:**
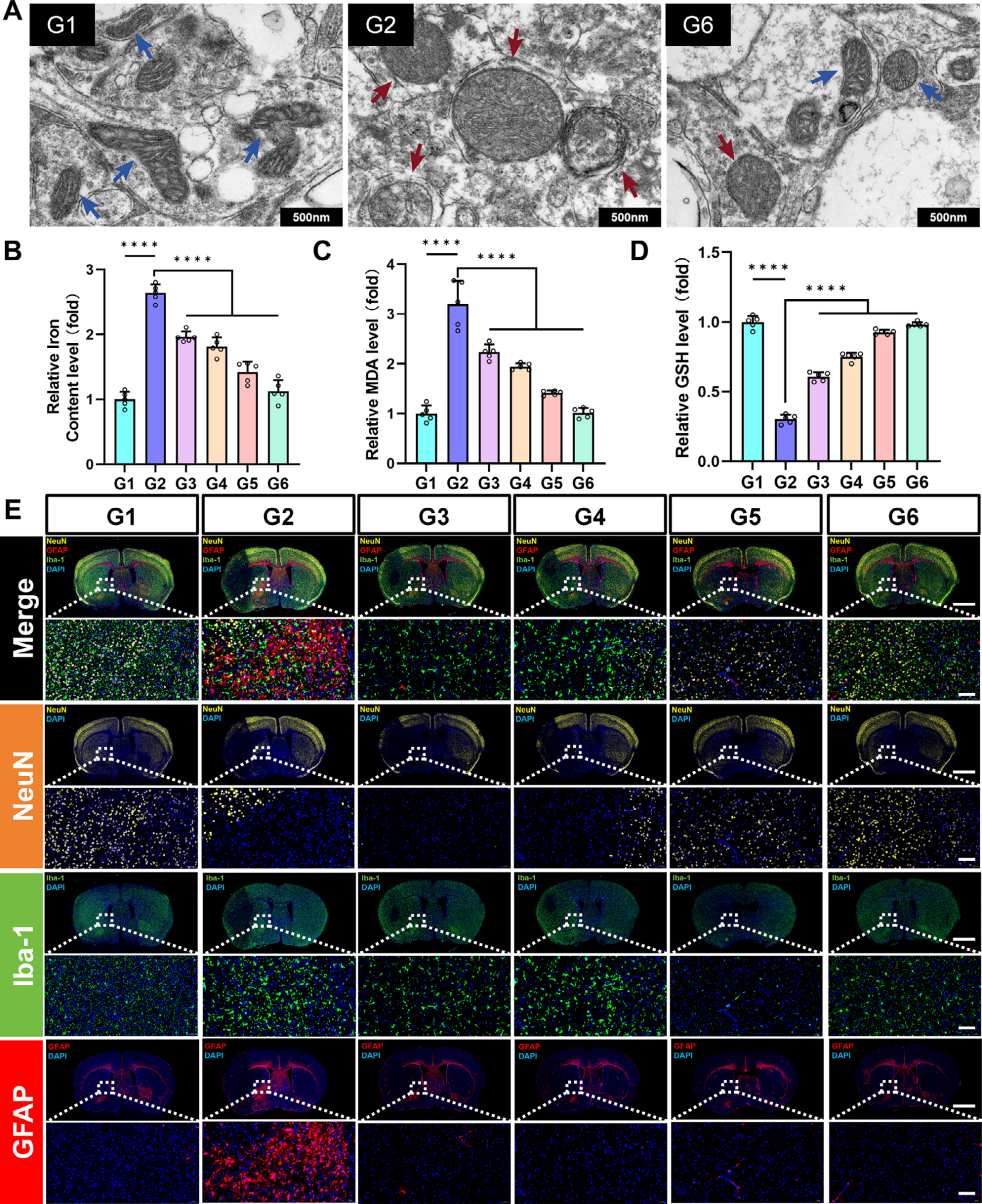
The inhibitory effect of RFP on ferroptosis in tMCAO/R mice and its regulatory role in the inflammatory microenvironment. A) Representative TEM images of mitochondrial morphology in the penumbra of the brain of different treatment groups of mouse. Blue arrows indicate normal mitochondria, while red arrows indicate abnormal mitochondria (scale bar, 100 nm). B–D) Detection of total iron content (B), MDA (C), and GSH (D) in the penumbra of the brain in mice from different treatment groups. Results are reported as means ± SD, n = 5, ^****^ *p* <0.0001. E) Immunofluorescence staining images of neuronal cells (NeuN), microglia (Iba‐1), and astrocytes (GFAP) in the brains of mice from different treatment groups (scale bar, 2 mm and 100 µm respectively).

The above in vitro findings prompted us to further explore the therapeutic efficacy of RFP in vivo. We established a tMCAO/R mouse model by inducing 1.5 h of transient cerebral ischaemia followed by reperfusion in C57 mice to simulate the pathophysiological process of CIRI. After removing the suture, we administered NPs via tail vein injection for intervention therapy (**Figure** **4**A). First, this study evaluated the blood‐brain barrier penetration and lesion targeting capability of RFP NPs in vivo. Using IVIS fluorescence imaging, we systematically examined intracranial fluorescence signals in three experimental groups: sham mice treated with RFP@Cy5.5, tMCAO/R mice treated with non‐targeted FP@Cy5.5, and tMCAO/R mice treated with targeted RFP@Cy5.5. The results showed distinct spatiotemporal distribution patterns of fluorescence signals among the groups. In the Sham group, the cerebral signal peaked at 4 h post‐injection and gradually declined, with only weak signals detectable at 16 and 24 h (Figure 4B). This indicates limited accumulation and rapid clearance of NPs in normal brain tissue with an intact blood‐brain barrier. The FP@Cy5.5‐treated tMCAO/R group also reached peak signal at 4 h, but the signal decayed more slowly than in the Sham group, maintaining relatively high levels from 8 to 16 h (Figure 4B). This suggested that blood‐brain barrier disruption facilitates passive retention of NPs in the brain, though the lack of specific targeting in FP@Cy5.5 limited its accumulation efficiency. In contrast, the RFP@Cy5.5‐treated tMCAO/R group demonstrated significant active targeting, reaching peak signal as early as 2 h post‐injection and maintaining high fluorescence levels throughout the 24 h observation period, indicating rapid accumulation and prolonged retention (Figure 4B). Throughout the observation period, the RFP@Cy5.5‐treated tMCAO/R group consistently showed the highest cerebral fluorescence intensity, followed by the FP@Cy5.5‐treated tMCAO/R group, while the sham group displayed the lowest signals (Figure S18, supporting information). These results demonstrate that cRGD peptide‐mediated active targeting significantly enhances both blood‐brain barrier penetration efficiency and intracranial accumulation of NPs. Ex vivo brain imaging at 24 h further validated these findings (Figure 5C; Figure S19, supporting information). Compared to the FP@Cy5.5‐treated tMCAO/R group, the RFP@Cy5.5‐treated tMCAO/R group exhibited stronger fluorescence signals at the whole‐brain level. More importantly, semi‐quantitative analysis revealed a significantly higher ischemic‐to‐contralateral hemisphere signal ratio in the targeted RFP@Cy5.5‐treated tMCAO/R group than in the non‐targeted FP@Cy5.5‐treated tMCAO/R group (Figure S20, supporting information). These results indicate that cRGD‐modified RFP NPs synergistically combine passive accumulation at the lesion site with receptor‐mediated active targeting to achieve efficient delivery to cerebral ischemic regions. Specifically, cRGD not only facilitated blood‐brain barrier penetration of RFP NPs but also enhanced active targeting and accumulation by binding to integrin αvβ3 receptors highly expressed in ischemic areas.^[^ ^40^ ^]^ In summary, RFP provides an effective strategy for precise targeted treatment of cerebral ischemia‐reperfusion injury.

### 2.5 Evaluation of the Therapeutic Effects of RFP with Antioxidant and Anti‐Ferroptosis Activity in tMCAO/R Mice

Subsequently, we subjected the mice in each group to different intervention methods, and performed Longa neurological function scoring and pathological examination 24 h later. The results showed that compared to the sham‐operated group (G1), the tMCAO/R group treated with saline injection (G2) exhibited severe neurological dysfunction, manifested as impaired spontaneous motor function (Figure 4D). In contrast, tMCAO/R mice treated with RF (G3), RP (G4), FP (G5), and RFP (G6) exhibited varying degrees of neurological functional recovery, with the RFP group (G6) showing the most significant improvement. Through TTC staining and quantitative analysis of infarct volume, it was found that treatment with different NPs reduced the infarct volume in tMCAO/R mice to varying degrees. Among them, RFP showed the most significant salvage effect on the ischemic penumbra, reducing the infarct volume to less than 10% of the total brain volume (Figure 4E,F; Table S2, Supporting Information). These results indicated that RFP effectively protected neurons, rescued the ischemic penumbra, and improved behavioral function in mice. We next continued to explore the neuroprotective mechanisms of RFP in brain tissue. Using the DHE probe, we measured the levels of ROS in the brains of mice from each group. Compared to the sham surgery group (G1), the tMCAO/R group (G2) exhibited a significant increase in brain ROS levels. In contrast, all treatment groups showed reduced brain ROS levels, with the RFP group (G6) demonstrating the most pronounced effect, indicating that RFP possesses superior antioxidant capacity in vivo (Figure 4G,H). Also, we evaluated the expression of key ferroptosis proteins in the brain. Immunofluorescence staining (IF) showed that different NPs had different effects on ferroptosis inhibition. RFP treatment significantly increased the expression levels of FTH1 and GPX4 in the brain and decreased the expression level of TfR1 (Figure 4G,I–K). We observed morphological changes in mitochondria using a transmission electron microscope (**Figure** **5**A) and found that mitochondria in the brain cells of tMCAO/R group (G2) mice were significantly damaged, as manifested by swelling, increased membrane density, and abnormal ridge structure. Mitochondrial morphology in the ischemic penumbra of mice treated with RFP (G6) was restored. Biochemical testing further confirmed that all treatment groups (especially the RFP group) significantly reduced iron levels and MDA levels in the ischaemic hemisphere while increasing GSH levels (Figure 5B–D). The above results proved that RFP, as a multifunctional brain‐targeted liposome loaded with Pt‐Ti_3_C_2_ nanoenzyme (SOD/CAT‐like enzyme activity) and Fer‐1 (specific ferroptosis inhibitor), exhibited excellent brain protection through antioxidant, anti‐ferroptosis, and mitochondria protection pathways.

### 2.6 Regulation of the Inflammatory Microenvironment by RFP

The large amount of ROS produced by oxidative stress and the DAMPs released by neurons undergoing ferroptosis can strongly drive the activation of surrounding glial cells (especially microglia and astrocytes), leading to a cascade of inflammatory responses.^[^ ^41^ ^]^ These two types of cells constitute the core of the inflammatory microenvironment and exacerbate neuronal damage. In Figure 5E, the IF results showed that there was significant infiltration and activation of microglia and astrocytes on the ischaemic side in the tMCAO/R group (G2). Specifically, the number of GFAP‐positive astrocytes significantly increased, with cell processes becoming thicker, longer, and more branched and dense. The number of Iba‐1‐positive microglia also increased dramatically, with cell bodies becoming larger and rounder, and processes becoming shorter, thicker, fewer, or even disappearing. Treatment with NPs can reverse the above abnormal changes to varying degrees and inhibit neuroinflammation. Among them, RFP showed superior anti‐inflammatory ability, achieving better therapeutic effects by regulating the pro‐inflammatory microenvironment.

### 2.7 Treatment Mechanism of RFP Based on Multi‐Omics Analysis

**Figure 6:**
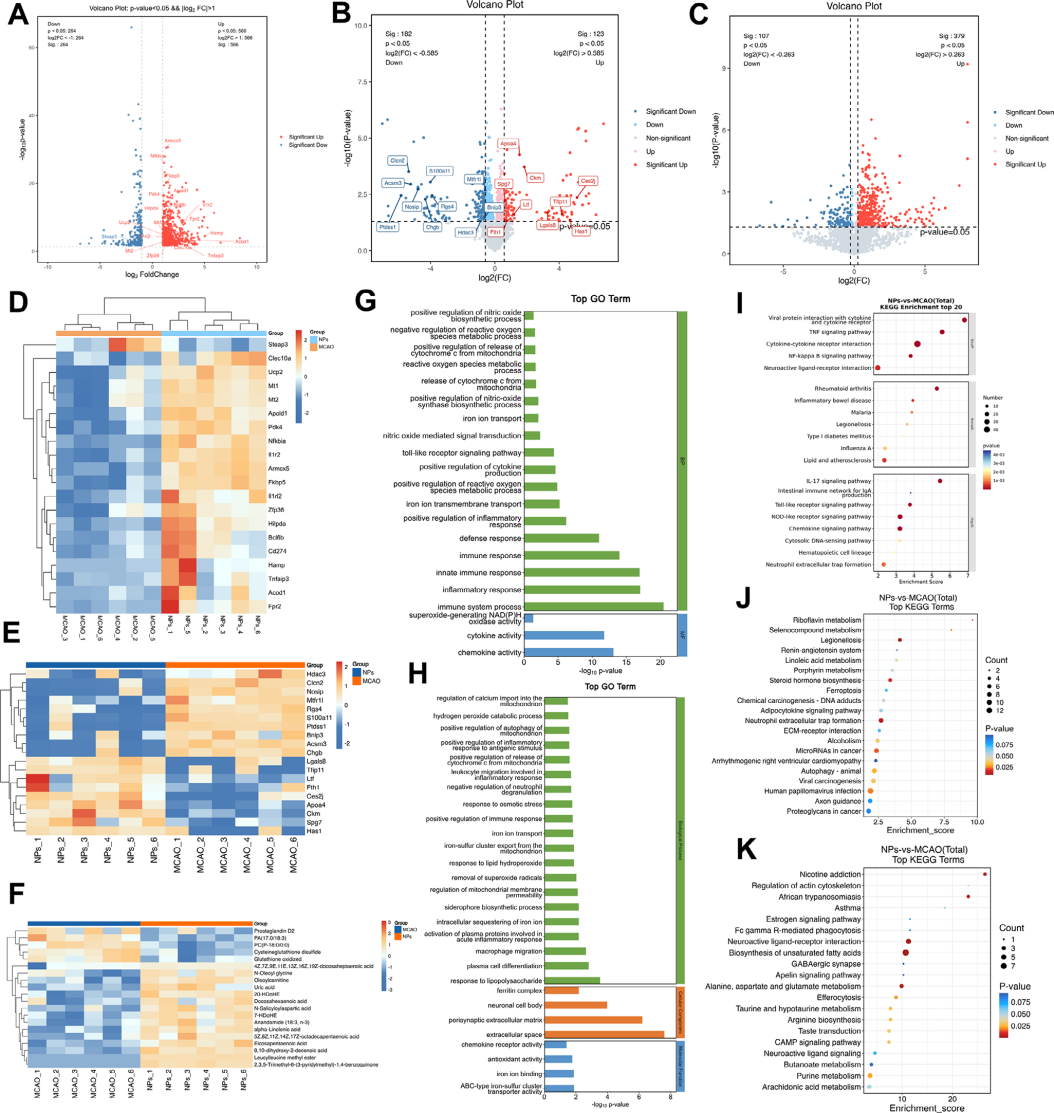
Analysis of transcriptomic, proteomic, and metabolomic sequencing results from brain tissue of tMCAO/R model mice and RFP treatment group mice. A) Volcano plot of DEGs between the two groups. B) Volcano plot of DEPs between the two groups. C) Volcano plot of differential metabolites between the two groups. D) Heatmapof DEGs between the two groups. (E) Heatmap of DEPs between the two groups. F) Heatmap of differential metabolites between the two groups. G,H) GO enrichment analysis results for DEGs (G) and DEPs (H) between the two groups. I–K) KEGG enrichment analysis results for DEGs (I), DEPs (J), and differential metabolites (K) between the two groups.

**Figure 7:**
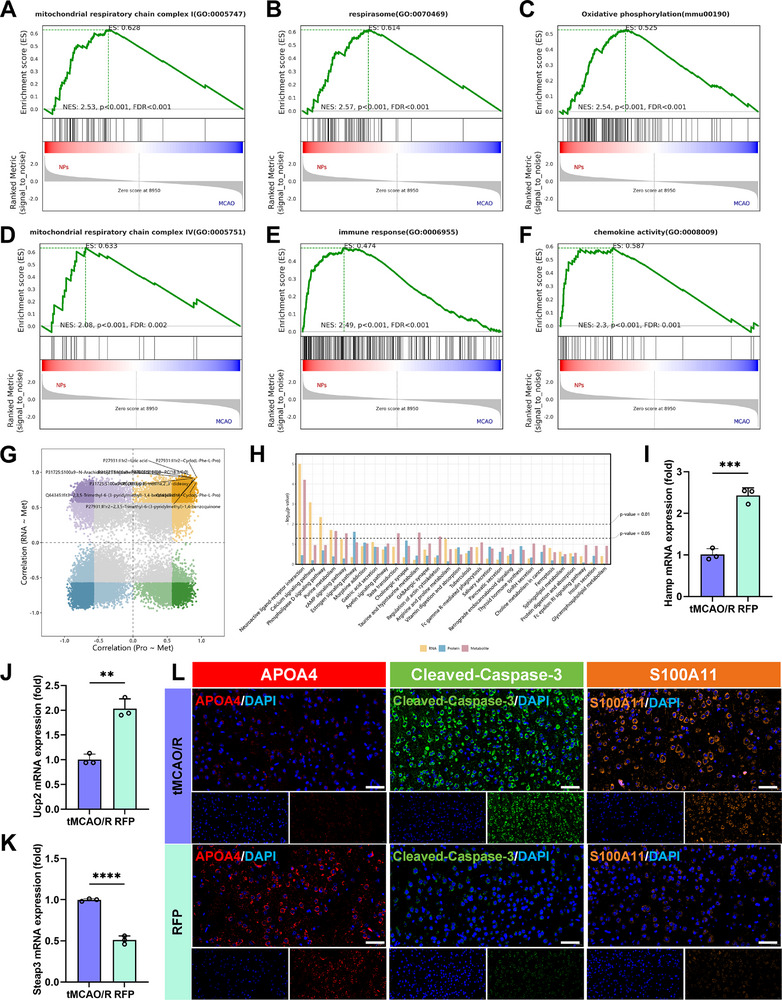
GSEA analysis results of transcriptome sequencing and combined multi‐omics analysis results of brain tissue from tMCAO/R model mice and RFP treatment group mice. A–F) Transcriptome sequencing GSEA analysis results. G) Quadrant diagram showing the correlation between transcriptomic, proteomic, and metabolomic sequencing results. H) Combined KEGG enrichment analysis results of transcriptomics, proteomics, and metabolomics sequencing results. I–K) Results of mRNA expression levels of Hamp (I), Ucp2 (J), and Steap3 (K) in brain tissue from each group of mice. Results are reported as means ± SD, n = 3. L) Representative images of immunofluorescence staining of APOA4, Cleaved‐Caspase‐3, and S100A11 in brain tissue from each group of mice (scale bar, 40 µm). ^**^ *p* <0.01, ^***^ *p* <0.001, ^****^ *p* <0.0001.

Through multi‐omics integration analysis (including transcriptomics, proteomics, and metabolomics) of the ischemic side brain tissue of tMCAO/R group mice and RFP NPs treatment group mice, this study systematically revealed the neuroprotective mechanism of RFP. Volcano plot analysis showed that the RFP NPs treatment group exhibited significant differences from the tMCAO/R control group in all three omics dimensions (**Figure** **6**A–C). Transcriptome sequencing revealed 830 differentially expressed genes between the tMCAO/R group and the RFP NPs treatment group. Compared to the tMCAO/R group, the expression levels of Hamp and Hilpda, which are genes related to ferroptosis inhibition, were significantly higher in the brain tissue of the RFP NPs group. Meanwhile, the expression level of Steap3, which is a gene related to ferroptosis promotion, was significantly lower. Notably, the expression levels of Ucp2, Mt1, and Mt2, which are related to antioxidant and mitochondrial homeostasis regulation, were significantly increased. Several inflammation‐related genes also underwent significant changes (Figure 6A). Proteome sequencing revealed 305 differentially expressed proteins between the two groups. The expression levels of the ferroptosis protein Fth1, the antioxidant protein ApoA4, and the mitochondrial homeostasis regulatory protein Ckm were significantly increased. Meanwhile, the expression levels of proteins such as Acsm3 and Mtfr1l were significantly decreased (Figure 6B). Metabolome sequencing identified 486 metabolites whose levels changed after treatment with RFP NPs. Several protective metabolites, such as DHA, significantly increased, while harmful metabolites, such as oxidized glutathione, significantly decreased (Figure 6C). Heat map analysis further confirmed that RFP NP treatment synergistically reversed ferroptosis, suppressed oxidative stress, maintained mitochondria homeostasis, and regulated neuroinflammation (Figure 6D–F). GO enrichment analysis showed that differentially expressed molecules were significantly enriched in pathways related to ferroptosis, oxidative stress, mitochondrial homeostasis, and immune inflammation, indicating that RFP NPs exerted therapeutic effects on tMCAO/R model mice through these pathways (Figure 6G,H). Consistent with the above findings, KEGG enrichment analysis also showed that differentially expressed molecules were significantly enriched in the above‐mentioned related pathways (Figure 6I–K; Figure S21, Supporting Information). Transcriptome GSEA enrichment analysis further corroborated the activation of mitochondrial energy metabolism pathways and the regulation of immune inflammation pathways in the RFP NPs treatment group (**Figure** **7**A–F; Figure S22, Supporting Information). Subsequently, through a combined analysis of three omics, we constructed a synergistic interaction network for RFP NPs (Figure 7G). KEGG pathway enrichment analysis revealed that treatment with RFP NPs resulted in significant alterations in multiple biological pathways at the RNA, protein, and metabolite levels. Among these, alterations in pathways related to ferroptosis, oxidative stress, mitochondrial function, and immune inflammation were particularly prominent (Figure 7H). This suggested that RFP NPs may exert therapeutic effects by intervening in injury through the aforementioned mechanisms. Based on these multi‐omics findings, we have revealed the potential mechanism of action of RFP NPs in interfering with the pathological process of CIRI at the multi‐molecular level.

To verify the transcriptome data, we performed qRT‐PCR to measure the mRNA expression levels of Hamp, Ucp2, and Steap3 in the ischemic cerebral tissue of tMCAO/R model mice and RFP NPs‐treated mice. The results showed that RFP treatment significantly upregulated the mRNA expression of Hamp and Ucp2, while downregulating Steap3 (Figure 7I–K). These changes were consistent with the RNA sequencing data and collectively suggest that RFP plays an important role in alleviating ferroptosis and oxidative stress. Hamp encodes hepcidin, and its increased expression inhibits the iron‐export function of ferroportin 1 (FPN1). This reduction in iron export limits intracellular free iron accumulation, thereby blocking the iron‐dependent lipid peroxidation cascade, which is a key mechanism in suppressing ferroptosis.^[^ ^42^, ^43^ ^]^ Ucp2, a member of the mitochondrial uncoupling protein family, not only lowers mitochondrial membrane potential and reduces ROS production but also maintains mitochondrial structural and functional integrity by regulating iron homeostasis within the mitochondrial matrix.^[^ ^44^ ^]^ Conversely, downregulation of Steap3, an iron reductase, inhibits lysosomal iron release and intracellular iron recycling, thereby reducing triggers of ferroptosis and acting synergistically with Hamp‐mediated iron metabolism regulation.^[^ ^45^ ^]^ Taken together, these gene expression changes demonstrate that RFP alleviates ferroptosis and oxidative stress at the source by regulating key iron metabolism genes (Hamp, Steap3) and the mitochondrial homeostasis gene Ucp2. This outcome is mechanistically linked to the functions of the Pt‐Ti_3_C_2_ nanozyme and Fer‐1 encapsulated within RFP.

At the protein level, we validated the proteomics findings through IF staining. The results showed a significant upregulation of APOA4 protein expression and a marked downregulation of Cleaved‐Caspase‐3 and S100A11 in the RFP‐treated group. This protein expression profile reveals a multi‐faceted protective mechanism of RFP treatment (Figure 7L; Figure S23, Supporting Information). As a member of the apolipoprotein family, APOA4 not only participates in the regulation of lipid metabolic homeostasis but also exhibits notable anti‐inflammatory activity. It mitigates the immune‐inflammatory response following ischemia‐reperfusion by suppressing the release of pro‐inflammatory cytokines and curbing microglial overactivation.^[^ ^46^ ^]^ The decreased expression of Cleaved‐Caspase‐3, a key executioner protease in the apoptosis pathway, directly indicates reduced cellular apoptosis. Given that both oxidative stress and ferroptosis can exacerbate brain injury by activating the Cleaved‐Caspase‐3‐mediated apoptotic pathway, the suppression of Cleaved‐Caspase‐3 suggests that RFP NPs can synergistically block multiple forms of cell death.^[^ ^47^ ^]^ S100A11, a calcium‐binding protein, is closely associated with inflammatory responses and blood‐brain barrier disruption. Its reduced expression demonstrates that RFP effectively inhibits the post‐ischemic immune‐inflammatory cascade and alleviates secondary brain tissue damage.^[^ ^48^ ^]^ These protein‐level changes further confirm that RFP enhances the expression of the anti‐inflammatory and cytoprotective protein APOA4 while suppressing the activation of the pro‐apoptotic and pro‐inflammatory proteins Cleaved‐Caspase‐3 and S100A11. This coordinated regulation of immune‐inflammatory responses and cell survival homeostasis complements the mechanisms of ferroptosis and oxidative stress inhibition revealed by transcriptomic analysis, together forming the core molecular network through which RFP exerts its neuroprotective effects.

### 2.8 In Vivo Biosafety Assessment of Nanoparticles

Biological safety assessment is an indispensable condition for the clinical application of novel nanomaterials. We conducted in vivo safety assessments on four types of NPs, which demonstrated good biocompatibility in major organs such as the heart, liver, spleen, lungs, and kidneys (Figure S24, Supporting Information). Histopathological examination confirmed that all examined organs had intact structures and showed no significant pathological damage such as inflammatory infiltration, necrosis, degeneration, or congestion. Additionally, serum biochemical indicators ALT and AST were within normal physiological ranges, further confirming that the NPs did not induce significant cardiac, hepatic, or renal dysfunction or tissue damage at the experimental dose and exposure period (Figure S25, Supporting Information). In summary, the NPs demonstrated excellent in vivo biosafety, providing an important guarantee for their biomedical applications.

Based on these findings, this study elucidates the potential molecular‐level mechanism by which RFP NPs intervene in CIRI, demonstrating potent therapeutic effects through synergistic regulation of ferroptosis, oxidative stress, and neuroinflammation. However, it is important to acknowledge a limitation in our current research. Although the tMCAO/R model employed here is the most widely used and highly reproducible system for investigating core pathophysiological mechanisms of CIRI, it does not fully recapitulate clinical scenarios involving thromboembolic occlusion and the administration of thrombolytic agents such as tPA, which represent critical components of stroke treatment in clinical practice. Future studies should utilize embolic stroke models combined with tPA thrombolysis to further validate the therapeutic efficacy and translational potential of the RFP nano‐platform under more clinically relevant conditions.

## 3 Conclusion

This study innovatively developed a multifunctional brain‐targeted nanoplatform (RFP NPs) co‐loaded with Pt‐based MXene nanoenzymes and ferroptosis inhibitors, which synergistically intervenes in the cascading pathological process of oxidative stress‐ferroptosis‐neuroinflammation to achieve multi‐effect treatment of cerebral ischaemia‐reperfusion injury. The core advantage of this nanoplatform lies in its integration of dual mechanisms of action: biomimetic catalysis and molecular inhibition. Pt‐Ti_3_C_2_ MXene efficiently scavenged reactive oxygen species with SOD/CAT‐like cascade enzyme activity. Density functional theory calculation revealed the catalytic nature of Pt‐Ti_3_C_2_ at the atomic scale. It explained that Pt modification endowed the material with excellent antioxidant capacity by optimizing substrate adsorption conformation, promoting interfacial charge transfer, and achieving ultra‐low energy barrier reaction kinetics. Ferrostatin‐1 specifically blocks the lipid peroxidation‐driven ferroptosis pathway. The synergistic action of the two significantly inhibits the excessive activation of glial cells by reducing the release of damage‐related molecular patterns, thereby reshaping the microenvironment of the ischemic brain area. Multi‐omics analysis further systematically elucidated the synergistic therapeutic effects of this nanoplatform through synchronous regulation of key ferroptosis pathways, enhancement of antioxidant defence, and inhibition of immune inflammatory networks. In conclusion, this study provides a new paradigm for overcoming the limitations of single‐target therapy and proposes a new strategy for the treatment of cerebral ischaemia‐reperfusion injury.

## 4 Experimental Section

### Synthesis of Ti_3_C_2_ MXene

Ti_3_C_2_ MXene was prepared using a fluoride salt/strong acid etching method. Ti_3_C_2_ powder (2.0 g) was slowly added to a mixture of LiF (3.2 g) and 9 m HCl (40 mL), stirred magnetically at 35 °C for 40 h, then centrifuged and washed to neutrality (pH 6) before being sonicated to obtain a colloidal solution of single‐ or few‐layer Ti_3_C_2_. Further size control was achieved through ice bath ultrasonic treatment (600 W, intermittent mode: 10s on/5s off, for 3 h), followed by freeze‐drying to obtain small‐sized Ti_3_C_2_ powder.

### Synthesis of Pt‐Ti_3_C_2_ MXene Nanoenzymes

Dispersion of 100 mg Ti_3_C_2_ in water, followed by sequential addition of sodium citrate solution (50 mg/10 mL) and chloroplatinic acid solution (100 mg/10 mL), stirred overnight, then centrifuged (12,000 rpm, 10 min), washed three times with water, and set aside for later use.^[^ ^36^, ^37^ ^]^

### Synthesis of Each Group of Liposomes

DSPC, DSPE‐PEG2000, and cholesterol were dissolved in a chloroform:methanol (2:1) mixed solvent (Fer‐1 was added at this stage for the Fer‐1‐loaded system), and a lipid film was formed by rotary evaporation. PBS and Pt‐Ti_3_C_2_ Mxene hydrate were added after vacuum drying, followed by ultrasonic treatment, and then extruded sequentially through 0.4 and 0.2 µm polycarbonate membranes. For the targeted modification system, the carboxyl groups on the surface of the liposomes were activated with EDC/NHS for 2 h, washed, and then reacted with cRGDyK peptide (5 mg) in PBS overnight. Four types of liposomes were ultimately constructed: 1)RF, 2)RP, 3)FP, and 4)RFP. All formulations were validated by DLS and Zeta potential and stored at 4 °C.^[^ ^49^ ^]^

### Physico‐Chemical Characterisation

The morphology of nanomaterials and the encapsulation efficiency of liposomes were characterized using transmission electron microscopy (TEM, FEI Tecnai G2 F20) and scanning electron microscopy (SEM, ZEISS Sigma 300). Samples were observed after being coated onto carbon copper grids at a concentration of 0.1 mg mL^−1^ and dried. The crystal structure of Pt‐Ti_3_C_2_ was analysed using high‐resolution TEM (HRTEM) combined with selected area electron diffraction (SAED), and the lattice spacing was calibrated using fast Fourier transform. The elemental composition was determined quantitatively and mapped using energy dispersive X‐ray spectroscopy (EDS). The chemical valence and phase structure were confirmed by X‐ray photoelectron spectroscopy (XPS, Al Kα rays) and X‐ray diffraction (XRD, Cu Kα rays, 5°–90° scan) analysis. The molecular bonding and optical properties of each component were evaluated using Fourier transform infrared spectroscopy (FTIR, KBr pellets) and UV–vis spectroscopy (UV–vis, 200–500 nm). The particle size distribution and Zeta potential of liposomes were determined using dynamic light scattering (DLS, 632.8 nm wavelength). The radical scavenging ability of nanoenzymes was evaluated by electron spin resonance (ESR, 320–340 mT magnetic field scanning) and enzyme activity detection (SOD‐like activity: WST‐1 method at 450 nm; CAT‐like activity: 240 nm H_2_O_2_ decay; DPPH scavenging: 517 nm). The encapsulation rate of Fer‐1 was determined by centrifugation to separate free drugs, followed by UV quantification (280 nm) using a standard curve (y = 0.13733x + 0.05456, R^2^ = 0.99636). The in vitro drug release characteristics were measured using dialysis (MWCO 3.5–14 kDa, PBS pH 6.5 containing 0.1% Tween‐80).

### Cell Culture and OGD/R Model Building

HT22 cells were cultured in high‐glucose DMEM medium containing 10% foetal bovine serum and 1% dual antibiotics (37 °C, 5% CO_2_). To establish the OGD/R model, logarithmic‐phase cells were seeded at an appropriate density. When confluence reached ≈70%, the medium was replaced with sugar‐free EBSS medium. The cells were immediately placed in a three‐gas incubator (37 °C, 1% O_2_, 94% N_2_, 5% CO_2_) for 4 h of hypoxia incubation to simulate ischaemia. The medium was then rapidly replaced with high‐glucose DMEM complete medium containing serum and reoxygenated under normoxic conditions (37 °C, 5% CO_2_) for 24 h, yielding an OGD/R‐induced in vitro I/R model.

### Animal Grouping and tMCAO/R Model Building

Male C57BL/6 mice (6–12 weeks old) were purchased from Jiangxi Zhonghong Boyuan Biotechnology Co., Ltd. All animal experiments were approved by the company's Animal Welfare and Ethics Committee (Reference number: LL‐202410150006). After anaesthetising and immobilising the mice, a neck incision was made, the area was shaved and disinfected, and the left common carotid artery (CCA), external carotid artery (ECA), and internal carotid artery (ICA) were exposed using blunt dissection. The proximal ends of the ECA and CCA were ligated, and a reserve suture was placed at the distal end of the CCA. The ICA was clamped with a haemostatic clamp, and an oblique incision was made at the proximal bifurcation of the CCA. A suture plug was inserted and secured with the reserve suture. The haemostatic clamp was released, and the suture plug was further inserted ≈9–11 mm to occlude blood flow in the middle cerebral artery. After 90 min of ischaemia, the suture plug was removed to restore blood flow, and the wound was sutured. The sham surgery group only exposed the vessels. After 24 h of reperfusion, Longa neurological function scores were assessed, and those meeting the criteria were randomly divided into six groups.

### Cell Viability Assay

The CCK8 assay was performed to assess the safety of NPs on HT22 cells. HT22 cells were seeded at 5×10^3^cells/well in a 96‐well plate and cultured for 24 h to allow them to adhere. The medium was then replaced with a gradient concentration of NPs (0–400 µg mL^−1^), with five replicate wells and a medium blank control. After further incubation for 24 h, 10 µL of CCK8 solution was added to each well and incubated in the dark for 2 h. The absorbance was measured at 450 nm using a microplate reader.

### Cell Uptake Study

HT22 cells were seeded at 2 × 10⁴ cells/well in confocal culture dishes. After 24 h, the medium was replaced with medium containing RFP NPs and incubated for 30 min. After discarding the medium, cells were washed three times with PBS, fixed with 4% paraformaldehyde for 15 min, and permeabilized with 0.1% Triton X‐100 for 10 min. Nuclei were stained with DAPI, the cytoskeleton was labelled with phalloidin, and the endogenous Cy5.5 signal from RFP NPs was used for tracking and localisation. Results were recorded using a confocal microscope.

### Transwell Migration Assay

As previously reported, an in vitro BBB model was established using a Transwell system. bEnd.3 cells were seeded onto the polycarbonate membrane of Transwell inserts at a specific density. The cells were cultured for 7 consecutive days with fresh medium replaced every two days. Formation of an intact cellular monolayer was monitored using a transendothelial electrical resistance meter. TEER values were measured daily starting from the first day after seeding. Only when the TEER values stabilized above 200 Ω·cm^2^ was the cell layer considered to have formed a complete blood‐brain barrier model and used for subsequent permeability experiments. Non‐targeted FP@Cy5.5 and cRGD‐targeted RFP@Cy5.5 were separately dispersed in serum‐free medium and added to the upper chambers of the Transwell system. The lower chambers were filled with fresh medium. After 24 h of incubation at 37 °C, the filtrate was collected from the lower chambers. The fluorescence intensity of the filtrate was measured using a microplate reader to quantitatively analyze the amount of nanoparticles that crossed the BBB. All experiments were independently repeated five times. The translocation percentage of NPs was calculated by normalization against a control group that penetrated bare filter membranes. To evaluate neuronal uptake capability, HT22 cells were directly seeded at the bottom of the lower chambers. After cell attachment, the pre‐established BBB model Transwell inserts were placed above them to form a co‐culture system. Following a 24 h incubation with nanoparticle‐containing medium in the upper chambers, the Transwell inserts were removed. Uptake of Cy5.5‐labeled NPs by HT22 cells in the lower chambers was directly observed and imaged using confocal microscopy.

### Assessment of Intracellular ROS and Lipid Peroxidation Levels

After modelling cells by OGD/R and treating them with different NPs, discard the culture medium and wash with PBS. Then add either 10 µm DHE working solution or 5 µm Liperfluo working solution, and incubate at 37 °C in the dark for 30 min. Digest the cells with trypsin, wash with PBS, and prepare a single‐cell suspension using a filter. Fluorescence intensity was measured using a flow cytometer (Beckman CytoFLEX) at 10 000 cells per sample, and changes in average fluorescence intensity were analysed using FlowJo software. The experiment included a normal control group, an OGD/R model group, and four NPs treatment groups, each repeated independently three times.

### Assessment of Mitochondrial Membrane Potential in Cells

After modelling HT22 cells by OGD/R and NPs intervention, discard the culture medium, wash with PBS, add 5 µg mL^−1^ JC‐1 working solution, and incubate in the dark for 20 min (37 °C). After trypsin digestion, wash with buffer and prepare a single‐cell suspension. Flow cytometry was used to measure the fluorescence intensity of 10000 cells per sample, and FlowJo was used to analyse membrane potential changes.

### Assessment of Neuronal Apoptosis Levels

Applied the Annexin V‐FITC/PI double staining method combined with flow cytometry to assess apoptosis in HT22 cells. Treated cells were collected after trypsin digestion and washed twice with pre‐chilled PBS. Following the kit instructions, cells were resuspended in binding buffer (1 × 10⁶ cells/mL), and 5 µL of Annexin V‐FITC and 5 µL of PI staining solution were added. The mixture was incubated at room temperature in the dark for 15 min. Flow cytometry was performed using a dual‐channel detector, with 10000 cells collected per group. The apoptosis ratio was quantified using FlowJo software.

### Measurement of Intracellular GSH and MDA

To detect intracellular oxidative stress and ferroptosis‐related markers, commercialized kits were used for biochemical analysis. Cells were lysed and the suspension was collected, then the following methods were used: 1) DTNB method to determine GSH content (412 nm); 2) TBA method to determine MDA levels (532 nm). Absorbance values were measured with a microplate reader, and concentrations were calculated using a standard curve and standardized by protein content (BCA method).

### QPCR Testing

Cells were digested with pancreatin and cultured to 80% confluence, washed with PBS, and total RNA was extracted using TRIzol reagent. After digestion with DNase I, reverse transcription was performed using PrimeScript RT. Amplification was performed using the SYBR Green qPCR kit on a fluorescence quantitative PCR instrument. GAPDH was used as an internal reference to calculate the relative expression level of the target gene, and the experiment was repeated three times. The primers used for QPCR are listed in Table S3 (Supporting Information).

### Neurological Assessment

To assess neurological dysfunction in mice, blind evaluation was performed using the Longa score. The scoring criteria were as follows: 0 points (no neurological deficit symptoms); 1 point (left forelimb cannot be fully extended); 2 points (circling to the left); 3 points (falling to the left); 4 points (loss of consciousness). The scores for each mouse in each group were recorded, and the average was calculated.

### TTC Staining and Assessment of Cerebral Infarction Volume

After completing the behavioral assessment, the mice were euthanized, and their brains were removed and rapidly frozen at −20 °C for 20 min. Coronary sectioning was performed (2 mm thick), and the sections were immersed in a 2% TTC solution (incubated at 37 °C in the dark for 30 min). After flipping the staining, the sections were fixed with 4% paraformaldehyde. Normal brain tissue appeared brick red, while the infarct area appeared pale white. Images of both sides of the sections were captured using a camera, and the infarct volume percentage was calculated using ImageJ software.

### Assessment of Oxidative Stress Levels in Brain Tissue

To assess ROS levels in ischemic brain tissue, immunofluorescence analysis was performed using a DHE fluorescent probe. 10 µm frozen brain sections were prepared, fixed with 4% PFA for 15 min, washed with PBS, and incubated in 5 µM DHE solution (37 °C, 30 min) in the dark. The sections were sealed with anti‐fluorescence quenching mounting medium, and images were collected using a fluorescence microscope. ImageJ was used to quantify the average fluorescence intensity in the ischemic penumbra region.

### Measurement of GSH, MDA, and Iron Level in Brain Tissue

Brain tissue was prepared into a 10% tissue homogenate. GSH, MDA, and iron levels were detected according to the commercial kit protocol. Absorbance values were read using a microplate reader, and concentrations were calculated using a standard curve. BCA method was used to normalize to protein content.

### Mitochondrial Morphology Assessment

Tissue blocks of 1 mm^3^ were taken from the ischemic penumbra cortical tissue and prepared into ultrathin sections. After double staining with uranium acetate and lead citrate, the morphological characteristics of mitochondria were observed by transmission electron microscopy. The integrity of the cristae structure, matrix density, and degree of mitochondrial swelling were analysed in detail.

### Immunofluorescence Staining

Cell samples were fixed with 4% paraformaldehyde for 15 min and permeabilized with 0.3% Triton X‐100 for 20 min. Brain tissue sections were dewaxed and rehydrated, followed by antigen retrieval with sodium citrate buffer, and then permeabilized with 0.3% Triton X‐100 for 20 min. The samples were then blocked with 1% BSA for 30 min. Target protein primary antibody was added and incubated at 4 °C overnight. After thorough washing with DPBS, the corresponding secondary antibody was incubated. Nuclei were stained with DAPI for 5 min, and target protein expression was analysed using a fluorescence microscope.

### Assessment of In Vivo Biosafety and Toxicity

The main organs of mice, including the heart, liver, spleen, lungs, and kidneys, were removed. The organs were fixed with 4% paraformaldehyde, embedded in paraffin, and prepared into 5 µm sections for H&E staining. The histopathological changes were observed using an optical microscope system. Mice serum was collected and the ALT and AST levels in the serum were measured using a fully automated biochemical analyser.

### Multi‐Omics Analysis

Mouse brain tissues from tMCAO/R model group and NP‐treated tMCAO/R group (n = 6 per group) were subjected to transcriptome sequencing, proteomic profiling, and untargeted metabolomics. Differential molecules were identified using omics‐specific thresholds: 1) Transcriptomic differentially expressed genes (DEGs) with P‐value < 0.05 and fold change (FC) > 2; 2) Proteomic differentially expressed proteins (DEPs) with P‐value < 0.05 and FC ≥ 1.5 or ≤ 1/1.5; 3) Metabolomic differential metabolites (DMs) with P‐value < 0.05 and FC ≥ 1.2 or ≤ 1/1.2. Functional characterization included Gene Ontology (GO) enrichment, Kyoto Encyclopedia of Genes and Genomes (KEGG) pathway analysis, and Gene Set Enrichment Analysis (GSEA) for DEGs, DEPs, and differential metabolites. Cross‐omics integration through correlation networks, co‐expression clustering, and KEGG‐based pathway mapping was performed to elucidate NP‐mediated therapeutic mechanisms in cerebral ischemia‐reperfusion injury.

### Statistical Analysis

Statistical analysis of the data was performed using GraphPad Prism software (version 9.0). Results were expressed as mean ± standard deviation. Statistical significance was assessed using paired t‐tests, unpaired t‐tests, and one‐way analysis of variance (ANOVA). Significance levels were indicated by ^*^ *p* <0.05, ^**^ *p* <0.01, ^***^ *p* <0.001, and ^****^ *p* <0.0001, respectively.

## Conflict of Interest

The authors declare no conflict of interest.

## Supporting information



Supporting Information

## Data Availability

The data that support the findings of this study are available from the corresponding author upon reasonable request.
