# Supplementary material for: Neuroprotective Nanoplatform Integrating Antioxidant MXene Nanozymes and Ferroptosis Inhibitors for Targeted Therapy of Cerebral Ischemia‐Reperfusion Injury
Source: Adv Sci (Weinh). 2025 Nov 21;13(7):e16001. doi: 10.1002/advs.202516001 (PMC12866792; doi:10.1002/advs.202516001)
Supplement: Supplementary file 1 — Supporting Information [file ADVS-13-e16001-s001.pdf]

Supporting Information

Neuroprotective Nanoplatform Integrating Antioxidant MXene Nanozymes and Ferroptosis Inhibitors for Targeted Therapy of Cerebral Ischemia-Reperfusion Injury

Lu Wang, Chao Hou, Shuo Li, Lizhi Yang, Yiqun Lin, Sen Wang, Xiaohua Jia, Hui Hui*, Wen He* and Wei Zhang**

Chemicals and materials.

Titanium aluminum carbide precursor Ti_3AlC_2 powder (purity≥98%, 200 mesh) was purchased from Suzhou Beike Nano Technology Co., Ltd. (China). Lithium fluoride (LiF, AR grade,≥99%), platinum chloride hydrate ($H_2PtCl_6 \cdot xH_2O$, ≥99.95%), Ferrostatin-1 (Fer-1, purity >98%), cholesterol (AR grade, ≥95%), 1-ethyl-3-(3-dimethylaminopropyl)carbodiimide hydrochloride (EDC·HCl, purity ≥98%), N-hydroxy succinimide (NHS, purity ≥98%) and methanol (CH_3OH, AR grade, ≥99.5%) were purchased from Aladdin (China). Hydrochloric acid (HCl, 36.0–38.0%, AR grade) was purchased from Sinopharm Chemical Reagent Co., Ltd. (China). Sodium citrate (purity ≥98%) and chloroform ($CHCl_3$, HPLC grade) were purchased from Sigma-Aldrich Corporation (USA). Cyclic arginine-glycine-aspartic acid peptide (c(RGDyK), purity ≥95%) was purchased from Guotai Biotechnology Co., Ltd. (China). Near-infrared fluorescent dye Cy5.5 (purity ≥97%) was purchased from Chongqing Yusi Pharmaceutical Technology Co., Ltd. (China). Distearoylphosphatidylcholine (DSPC, purity ≥98%) was purchased from Shanghai Bide Pharmaceutical Technology Co., Ltd. (China).

1,2-Distearoyl-sn-glycero-3-phosphoethanolamine-N-[methoxy(polyethylene glycol)-2000] (DSPE-PEG-2000, purity ≥98%) was purchased from Shanghai McLean Biochemical Technology Co., Ltd. (China). Polycarbonate membrane filters with pore sizes of 0.4 μm and 0.2 μm were purchased from Whatman Company (UK). Ultra-pure water (resistivity ≥ 18.2 MΩ·cm) was purchased from Millipore Company (USA).

Reactive Oxygen Species Assay Kit (CA1420) and Mitochondrial Membrane Potential Assay Kit with JC-1 (M8650) were purchased from Solarbio (China). Liperfluo probe （L248） and Cell Counting Kit-8（CK04） were purchased from DOJINDO (Japan). Annexin V-FITC/PI apoptosis kit (AP101) was purchased from Multi Sciences (China). MDA detection kit (G4300/G4302), GSH detection kit (G4303), total iron ion detection kit (G4301) and BCA protein quantification detection kit (G2026) were all purchased from Servicebio (China). Anti-CD71 antibody (84766-4-RR, Proteintech), Anti-GPX4 antibody (67763-1-Ig, Proteintech), and Anti-Ferritin heavy chain antibody (11682-1-AP, Proteintech) were purchased from Proteintech. Anti-APOA4 antibody (GB111436), Anti-S100A11 antibody (GB111080), Anti-Cleaved-Caspase-3 antibody (GB11532), Anti-NeuN antibody (GB11138), Anti-Iba1 antibody (GB12105), and Anti-GFAP antibody (GB11096) were all purchased from Servicebio (China). 2,3,5-Triphenyltetrazolium chloride (TTC) and 4',6-Diamidino-2-phenylindole (DAPI) were purchased from Servicebio (China).

Density functional theory calculations.

The DFT calculations in this work were performed using the Dmol^3 package in the software of Materials Studio 2019. The exchange correlation effects were accounted for by using the generalized gradient approximation (GGA) of PBE employed for the exchanged-correlation functional together with the double-numerical quality basis set with polarization functions (DNP with 3.5 basis file). A global orbital cut off of 5.0 Å was adopted to improve the computational performance, and the core electrons were treated with Effective Core Potentials. DFT-D scheme for dispersion correction was adopted to describe the van der Waals interactions. The tolerances of the energy, gradient, and displacement convergence were 10^{-5} Ha (1 Ha = 27.212 eV), 0.002 Ha/Å, and 0.005 Å, respectively. The SCF tolerance was set as 10^{-5} Ha.

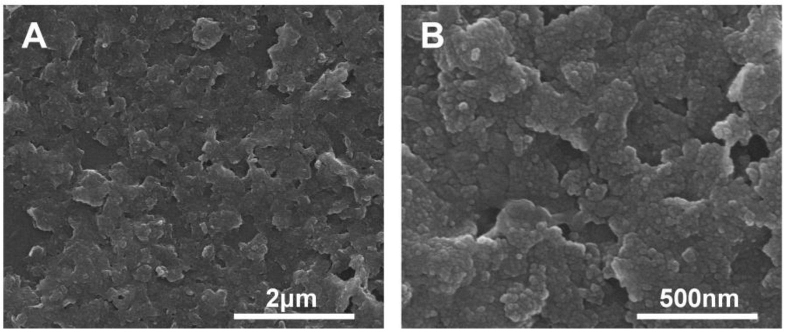

Figure S1. Representative SEM images of Pt-Ti_3C_2 (scale bar, 2 μm and 500nm respectively).

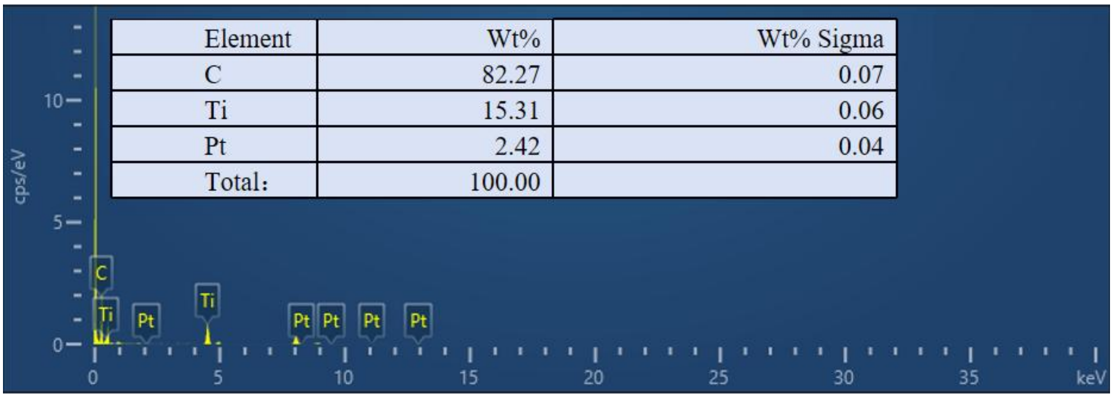

Figure S2. EDS spectrum of Pt-Ti_3C_2.

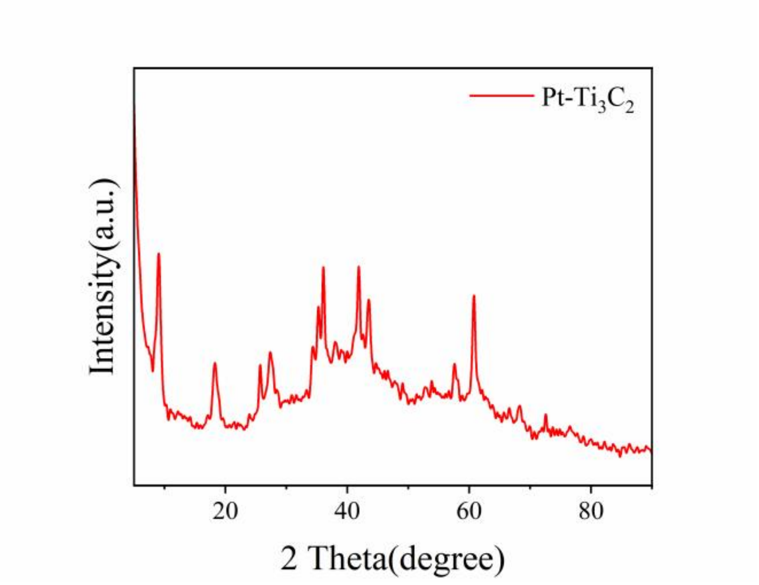

Figure S3. XRD pattern of Pt-Ti_3C_2.

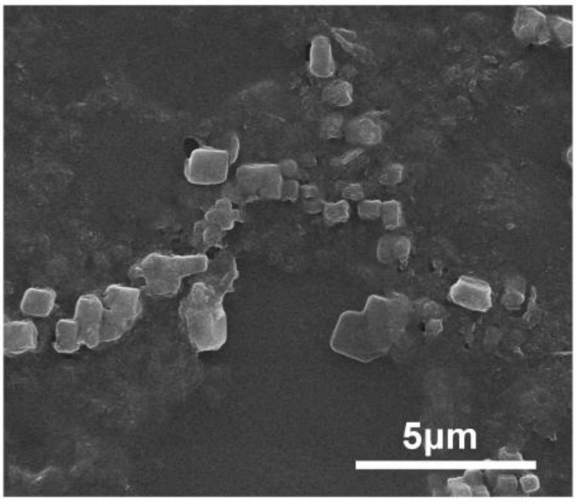

Figure S4. Representative SEM images of RFP (scale bar, 5μm).

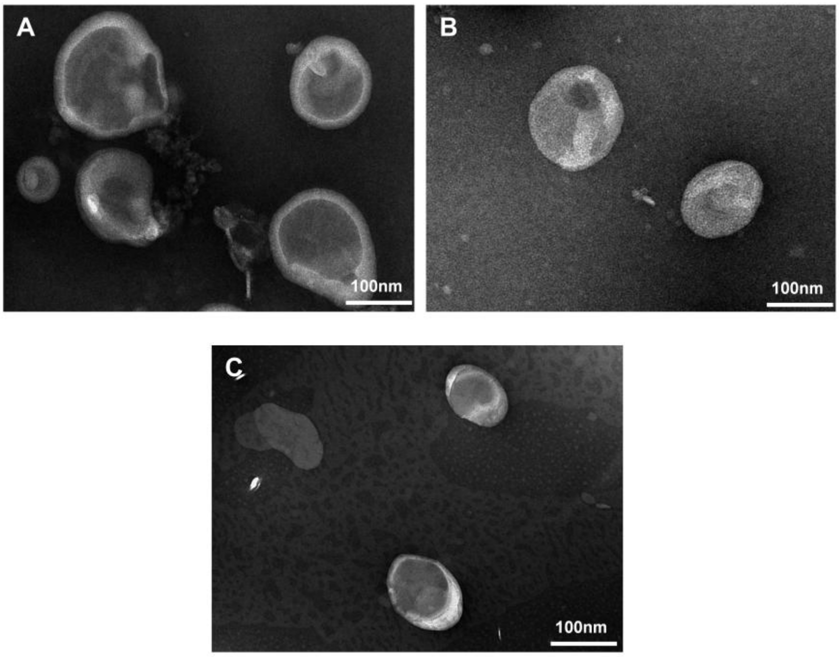

Figure S5. Representative TEM images of RF (A), RP (B) and FP (C) (scale bar, 100 nm).

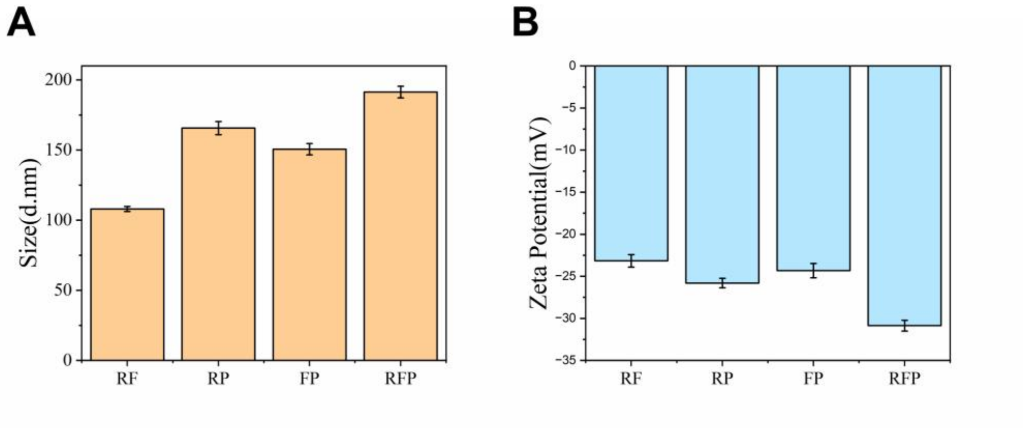

Figure S6. Particle size (A) and zeta potential (B) of different nano particles. Results are presented as means ± SD, n = 3.

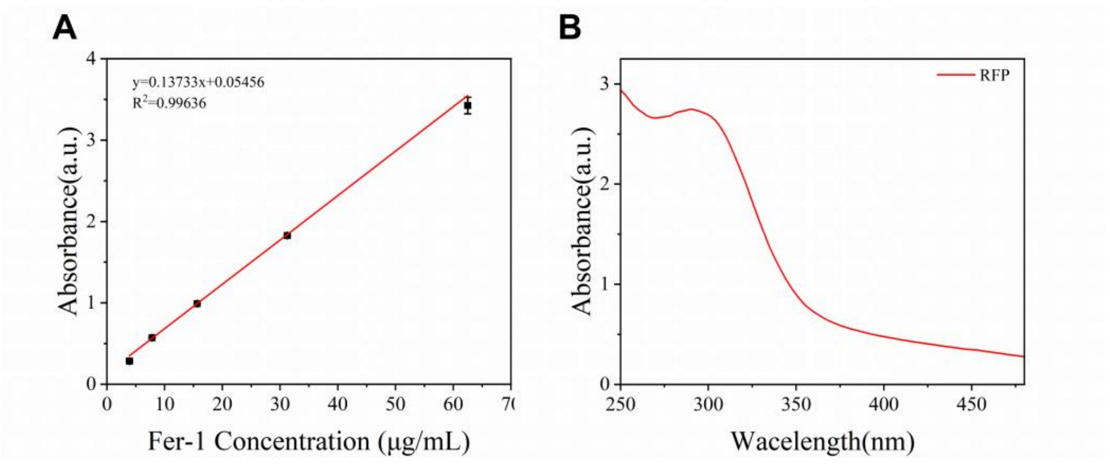

Figure S7. Calculation of encapsulation efficiency and drug loading efficiency of Fer-1 in RFP. (A) Standard curve (B) UV-Vis of RFP supernatant.

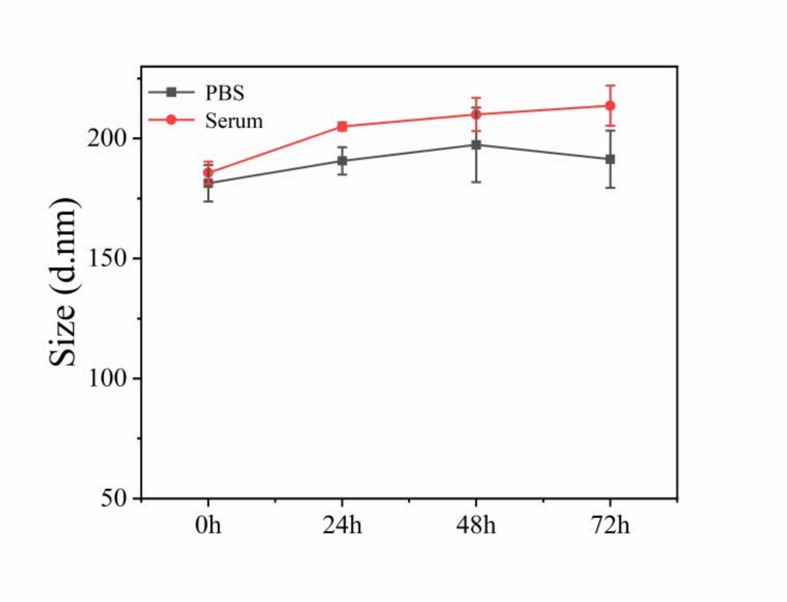

Figure S8. Changes in particle size of RFP in PBS and serum over time.

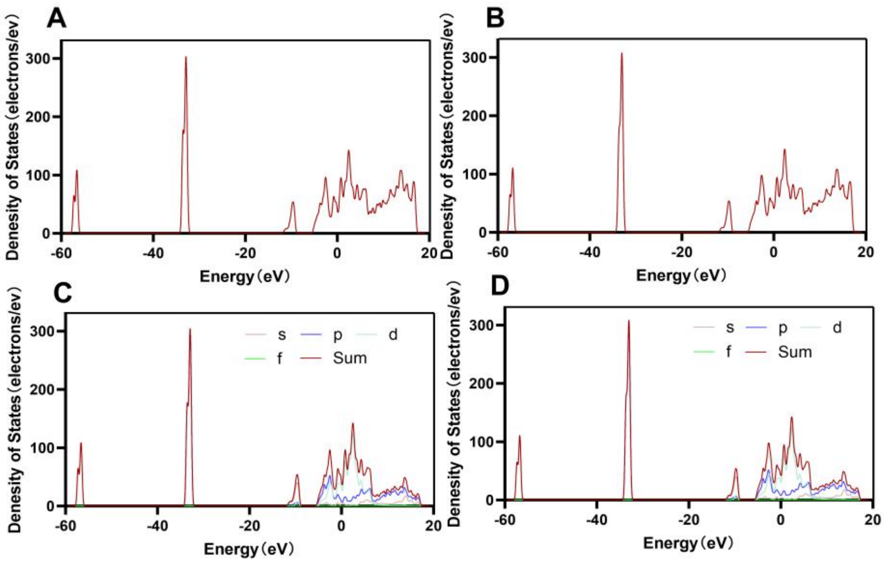

Figure S9. Comparison of the total density of states (DOS) and analysis of the partial density of states (PDOS) for Ti_3C_2 and Pt-Ti_3C_2. (A) DOS of Ti_3C_2. (B) DOS of Pt-Ti_3C_2. (C) PDOS of Ti_3C_2. (D) PDOS of Pt-Ti_3C_2.

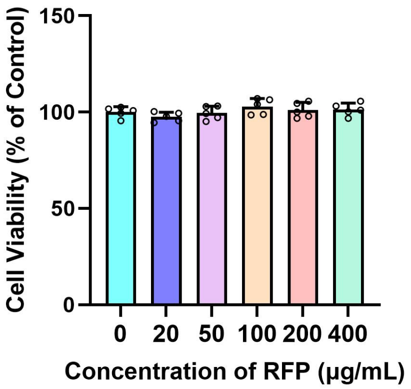

Figure S10. The cell viability test. The safety of RFP on HT22 cells, cells were incubated with RFP in different concentrations for 24 hours. Results are presented as means ± SD, n = 5.

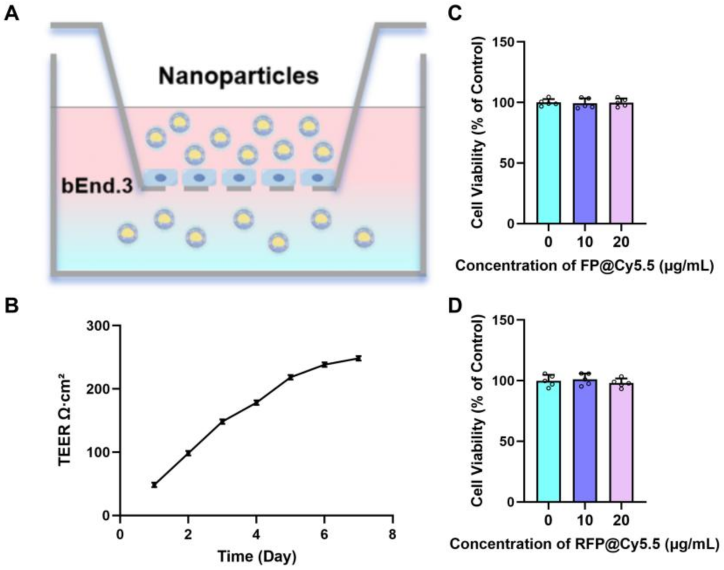

Figure S11. Establishment and characterization of an in vitro BBB model and evaluation of nanoparticle biocompatibility. (A) Illustration of the in vitro BBB transwell model. (B) TEER value of the in vitro BBB model from the first day to the seventh day. Results are reported as means ± SD, n = 3. (C-D) Cell viability of bEnd.3 cells after incubation with FP@Cy5.5 (C) and RFP@Cy5.5 (D) for 48 h. Results are

reported as means ± SD, n = 5.

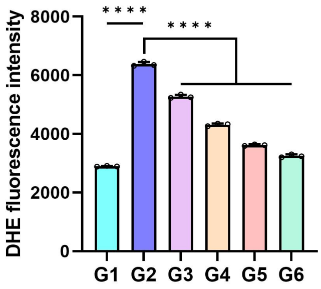

Figure S12. The semiquantitative results of DHE fluorescence intensity in HT22 cells with different treatments. Results are presented as means ± SD, n = 3, ****P < 0.0001.

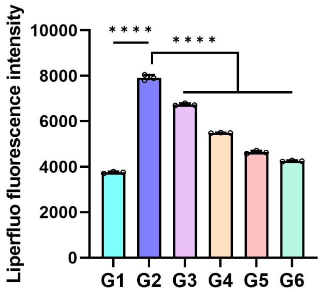

Figure S13. The semiquantitative results of Liperfluo fluorescence intensity in HT22 cells with different treatments. Results are presented as means ± SD, n = 3, ****P < 0.0001.

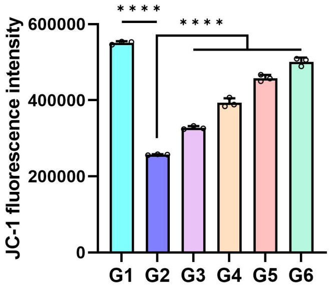

Figure S14. The semiquantitative results of JC-1 fluorescence intensity in HT22 cells with different treatments. Results are presented as means ± SD, n = 3, ****P < 0.0001.

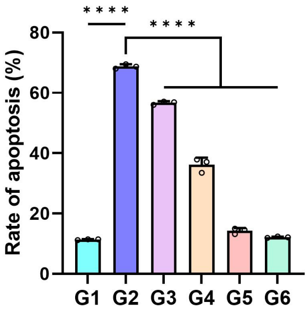

Figure S15. Quantitative analysis of apoptosis rate of each treatment group. Results are presented as means ± SD, n = 3. ****P < 0.0001.

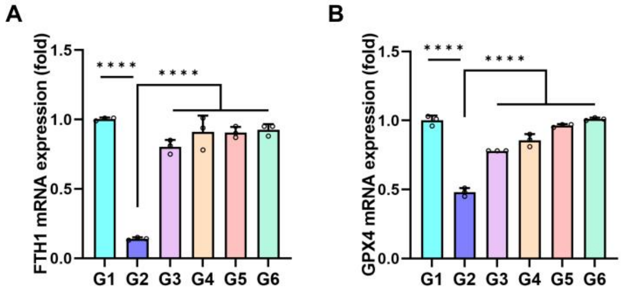

Figure S16. Results of mRNA expression levels of FTH1 (A) and GPX4 (B) in each group of cells. Results are reported as means ± SD, n = 3. ****P < 0.0001.

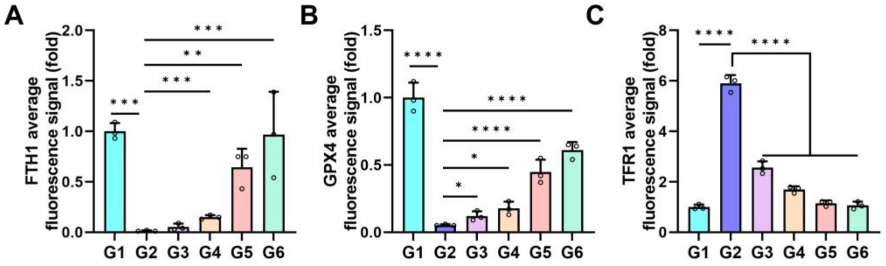

Figure S17. Semi-quantitative results of the fluorescence intensity in Figure 3K. (A) FTH1. (B) GPX4. (C) TFR1. Results are reported as means ± SD, n = 3. *P < 0.05, **P < 0.01, ***P < 0.001, ****P < 0.0001.

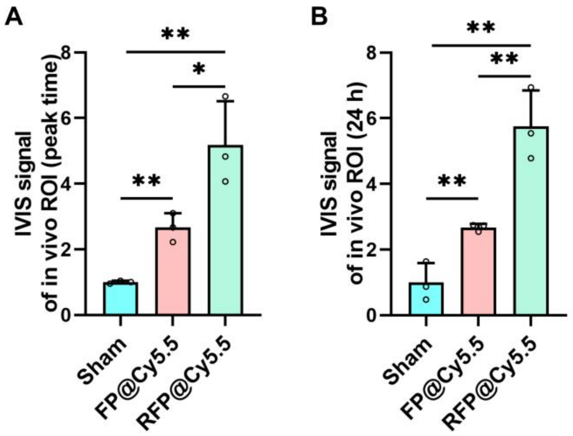

Figure S18. Semiquantitative analysis of IVIS signals for each group of mice. (A) Semiquantitative analysis of IVIS signals at peak time for each group of mice. (B) Semiquantitative analysis of IVIS signals in each group of mice after 24 hours. Results are reported as means ± SD, n = 3. *P < 0.05, **P < 0.01.

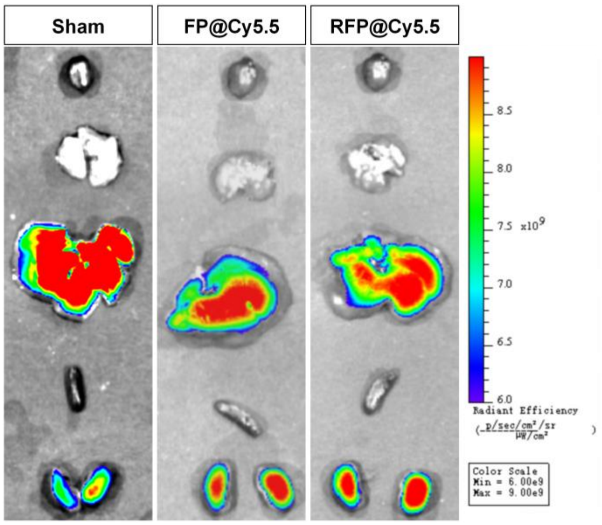

Figure S19. Ex vivo IVIS images of main organs after intravenous administration of FP@Cy5.5 or RFP@Cy5.5 for 24 h.

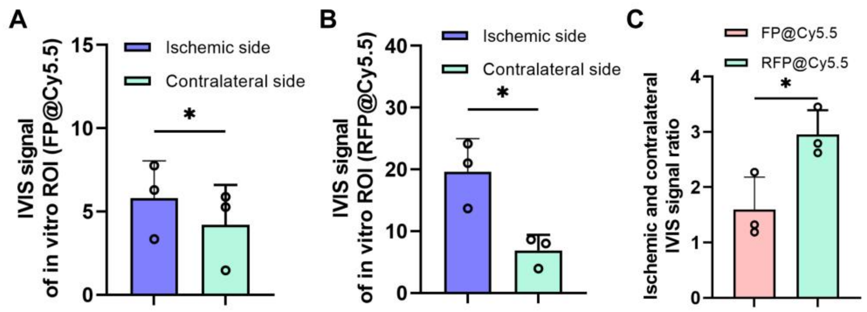

Figure S20. Semiquantitative analysis of signal intensity in the ischemic and contralateral hemispheres of ex vivo mouse brains. (A) Semiquantitative analysis of signal intensity in the ischemic and contralateral hemispheres of ex vivo mouse brains following FP@Cy5.5 injection. (B) Semiquantitative analysis of signal intensity in the ischemic and contralateral hemispheres of ex vivo mouse brains following RFP@Cy5.5 injection. (C) The signal ratio was significantly higher in the ischemic hemisphere of tMCAO/R mice treated with targeted RFP@Cy5.5 compared to those treated with non-targeted FP@Cy5.5. Results are reported as means ± SD, n = 3. *P < 0.05.

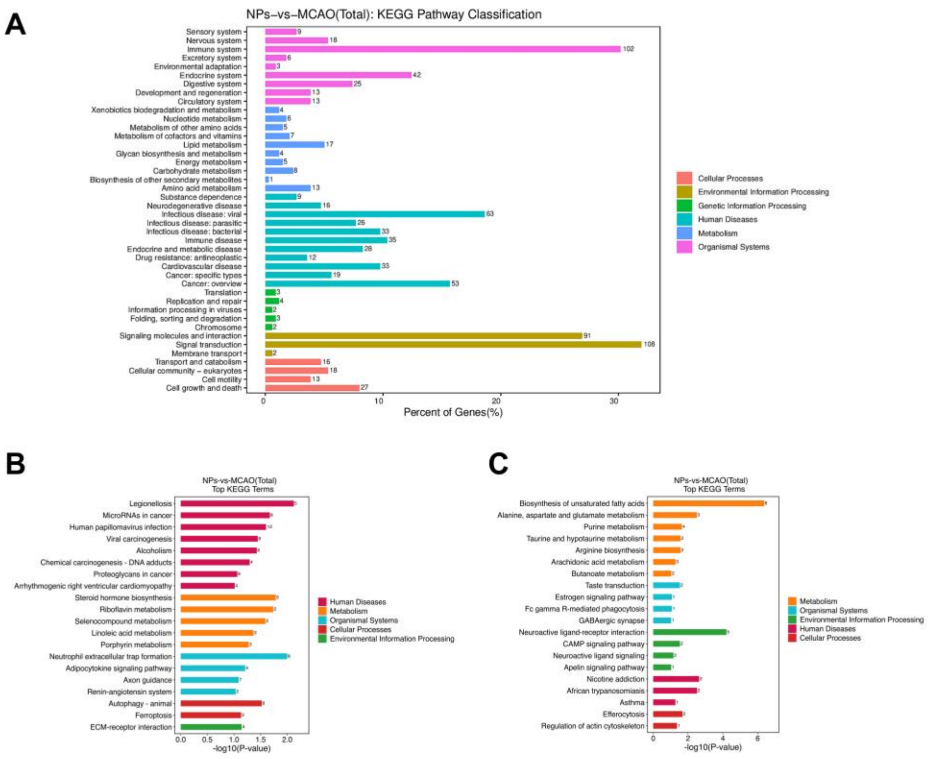

Figure S21. KEGG analysis of DEGs (A), DEPs (B), and differential metabolites (C) between the model group and the RFP treatment group.

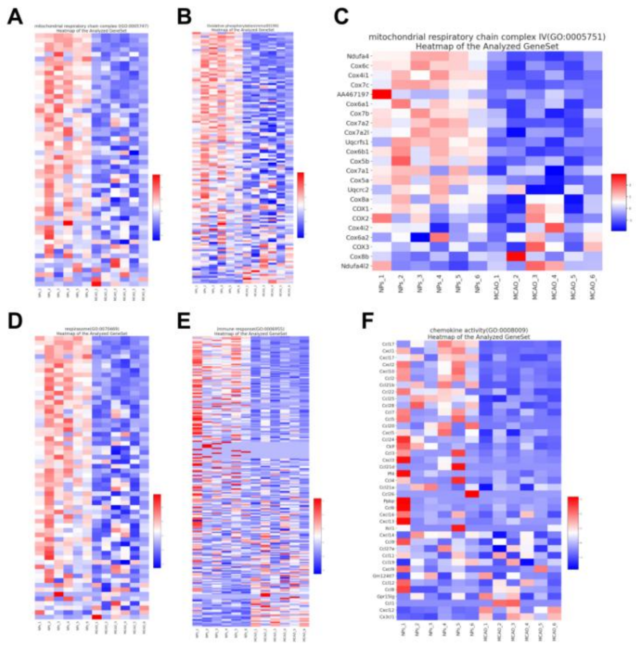

Figure S22. GSEA analysis of transcriptomic sequencing.

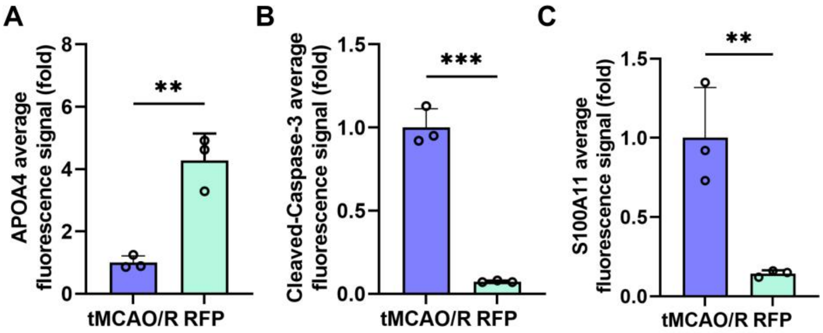

Figure S23. Semi-quantitative results of the fluorescence intensity in Figure 7L. (A) APOA4. (B) Cleaved-Caspase-3. (C) S100A11. Results are reported as means ± SD, n = 3. **P < 0.01, ***P < 0.001.

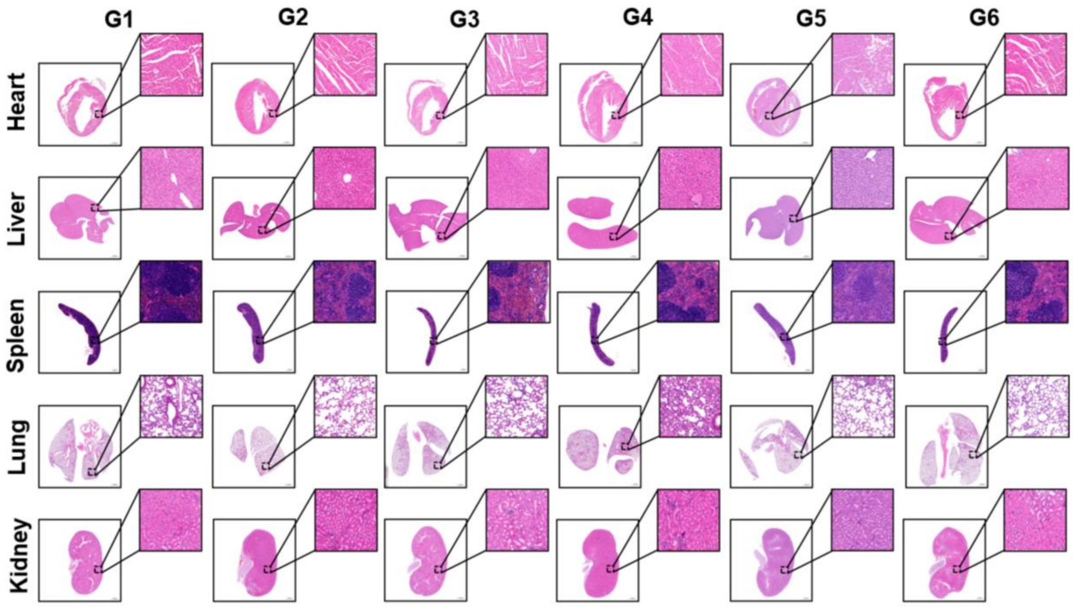

Figure S24. Comprehensive H&E staining images depicting complete views of the main organs.

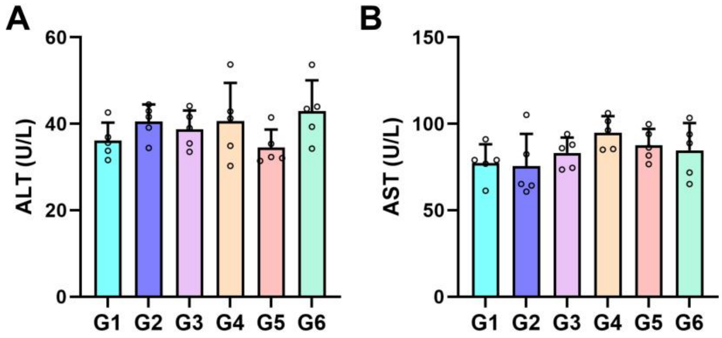

Figure S25. The level of ALT (A) and AST (B) in mouse after being treated with different nanoparticles. Results are reported as means ± SD, n = 5. ALT, alanine aminotransferase; AST, aspartate aminotransferase.

Table S1. Description of particle size and zeta potential of nanoparticles in each group. Results are reported as means ± SD, n = 3.

Nanoparticles	Particle size (d, nm)	Zeta potential (mV)
RF	108.00 ± 1.73	-23.17 ± 0.75
RP	165.67 ± 4.73	-25.80 ± 0.56
FP	150.67 ± 4.04	-24.33 ± 0.85
RFP	191.33 ± 4.16	-30.87 ± 0.64

Table S2. Quantitative analysis of infarct volume in various nanoparticle treatment groups. Results are reported as means ± SD, n = 6.

**Group**	**Infarct area (%)**
G2	48.83±3.60
G3	36.63±2.81
G4	33.38±3.64
G5	14.38±1.62
G6	6.20±1.32

Table S3. Primer Sequences Used for QPCR.

Primers names	Primers sequences (5'→3')
FTH1-Forward	CATCAACCGCCAGATCAACCT
FTH1-Reverse	GCAAAGTTCTTCAAAGCCACATC
tfr1-Forward	GGCAAGTAGATGGCGATAACAG
tfr1-Reverse	CAATAGCCCAAGTAGCCAATCA
Hamp-Forward	AGGGCAGACATTGCGATACC
Hamp-Reverse	GCAACAGATACCACACTGGGA
Ucp2-Forward	TGCGGTCCGGACACAATAG
Ucp2-Reverse	CAGTGTCGGGAAATGCTCAG
Steap3-Forward	ACCTGGTATGCCACATCTCAA
Steap3-Reverse	GGAACCCTAGCACCTGGAAT
